# Single-cell immune transcriptomics reveals an inflammatory-inhibitory set-point spectrum in autoimmune diabetes

**DOI:** 10.1172/jci.insight.199050

**Published:** 2025-11-25

**Authors:** Ivan I. Golodnikov, Elizaveta S. Podshivalova, Vadim I. Chechekhin, Anatoliy V. Zubritskiy, Alina A. Matrosova, Nikita A. Sergeev, Margarita D. Samsonova, Yaroslav V. Dvoryanchikov, Tatiana V. Nikonova, Ekaterina V. Bondarenko, Marina Yu. Loguinova, Yulia A. Medvedeva, Dmitry N. Laptev, Rita I. Khusainova, Ildar R. Minniakhmetov, Marina V. Shestakova, Natalia G. Mokrysheva, Ivan I. Dedov

**Affiliations:** 1 Endocrinology Research Centre, Moscow, Russia.; 2 Medical Research and Educational Institute, Lomonosov Moscow State University, Moscow, Russia.; 3 Federal Research Centre “Fundamentals of Biotechnology” of the Russian Academy of Sciences, Moscow, Russia.

**Keywords:** Autoimmunity, Endocrinology, Genetics, Autoimmune diseases, Diabetes, Expression profiling

## Abstract

Autoimmune diabetes encompasses rapidly progressive type 1 diabetes mellitus (T1D) and indolent latent autoimmune diabetes in adults (LADA), which represent distinct inflammatory set points along a shared autoimmune spectrum. Yet, the immunological mechanisms that determine these divergent inflammatory states remain unresolved. We performed single-cell RNA sequencing with paired T and B cell receptor profiling on over 400,000 PBMCs from patients with LADA, newly diagnosed T1D, and individuals acting as healthy controls. PBMC composition was comparable across cohorts, indicating that qualitative rather than quantitative immune differences underlie disease heterogeneity. In T1D, pan-lineage activation of NF-κB, EGFR, MAPK, and hypoxia pathways, coupled with a TNF-centered communication hub, enhanced MHC signaling, disrupted adhesion, and promoted systemic inflammation. LADA, by contrast, exhibited global suppression of NF-κB/EGFR activity, retention of moderate JAK/STAT tone, reinforced NK cell inhibitory checkpoints via HLA-C–KIR2DL3/3DL1 interaction, and stabilized CD8^+^ T cell synapses through HLA-C–CD8 binding, collectively restraining effector activation. Single-cell V(D)J analysis revealed multiclonal, patient-unique adaptive repertoires, emphasizing the primacy of signaling context over receptor convergence. These findings define autoimmune diabetes as an inflammatory-inhibitory set-point continuum, positioning the NF-κB/EGFR–JAK/STAT gradient and HLA-C–KIR axis as potential therapeutic targets to preserve residual β cell function.

## Abstract 2

Single-cell analysis shows that slower autoimmune diabetes has a calmer immune response, offering insight into why some patients lose insulin production more gradually.

## Introduction

Latent autoimmune diabetes in adults (LADA) is a form of autoimmune diabetes characterized by serological hallmarks typical of type 1 diabetes mellitus (T1D), including β cell autoantibodies and T cell–mediated insulitis, but with a notably delayed onset and slower decline in endogenous insulin secretion. Clinically, this indolent progression presents two significant dilemmas: (a) identifying individuals who would benefit from early immunomodulatory intervention and (b) preserving residual β cell function after the onset of autoimmune destruction (1, 2). A central mechanistic uncertainty remains unresolved: does LADA represent merely a milder phenotype on the autoimmune diabetes spectrum, or do specific immunoregulatory mechanisms actively restrain islet cytotoxicity, moderating disease severity (3)? In this study, we adopt the framework of autoimmune diabetes as a clinical and immunological spectrum, while adjusting for age to minimize confounding between LADA and new-onset T1D.

Peripheral immunophenotyping reveals largely overlapping distributions of major leukocytes between individuals with LADA and T1D. Specifically, frequencies of IFN-γ^+^CD4^+^ and CD8^+^ T cells, as well as CD4^+^ effector T cells, appear comparable across both groups. Like patients with T2D, patients with LADA had increased frequencies of CD4^+^ Tem and CD8^+^ Tem cells with respect to T1D (4). A monocyte subpopulation marked by SIGLEC1 and enriched for interferon-response genes was expanded in individuals with LADA and pediatric T1D, suggesting shared features of innate immune activation at disease onset (5). These findings suggest that subtle immunophenotypic differences are insufficient to explain the slower β cell destruction in LADA; qualitative distinctions in signaling, effector licensing, or checkpoint function are more likely responsible.

Emerging evidence from bulk transcriptomic studies supports this concept of qualitative divergence. Early analyses of patients with LADA have revealed heightened expression of proinflammatory genes in monocytes (6); RNA sequencing of peripheral neutrophils from newly diagnosed patients with LADA revealed marked enrichment of the NF-κB signaling pathway, as one of the top-ranked KEGG categories (7); and altered cytokine-cytokine receptor interaction signatures in PBMCs have been investigated by bulk RNA sequencing (8). However, bulk RNA sequencing inherently averages transcriptional signals across heterogeneous cell mixtures, thereby masking cell-specific transcriptional states and low-frequency pathogenic subsets. Additionally, comparative analyses directly involving newly diagnosed patients with T1D alongside LADA cohorts are rare, leaving substantial gaps in understanding precisely which molecular programs differentiate these autoimmune diabetes variants.

We hypothesized that the slower clinical progression of LADA results from selective attenuation of inflammatory signaling cascades, especially NF-κB and growth factor–related pathways (e.g., EGFR), while simultaneously maintaining sufficient JAK/STAT signaling to sustain chronic, low-grade autoreactivity (9–11). Single-cell RNA sequencing (scRNA-seq), combined with computational pathway inference tools, such as PROGENy, permits quantitative characterization of signaling activities at single-cell resolution, revealing functional heterogeneity otherwise obscured by bulk approaches. Such high-dimensional profiling is thus uniquely suited for identifying critical checkpoints that selectively restrain pathogenic effector cells in LADA but fail in rapidly progressing T1D.

Accordingly, we profiled 4.4 × 10^5^ peripheral-blood immune cells from individuals with LADA, individuals with newly diagnosed T1D, and matched individuals acting as healthy controls using 5′ scRNA-seq. We integrated transcriptional data, pathway activity scores, and cell-to-cell ligand-receptor interaction analyses to elucidate both cell-intrinsic and intercellular signaling programs underlying differential disease progression. By connecting clinical phenotypes to molecular immune circuitry, this study refines the conceptual framework of autoimmune diabetes pathogenesis and identifies actionable immunological checkpoints, such as the inhibitory HLA-C/KIR2DL3 axis and regulatory CLEC2D/KLRB1 interactions, as potential therapeutic targets for preserving residual β cell function in LADA.

## Results

### Baseline characteristics of the study population

Key demographic and anthropometric characteristics of the study participants are summarized in Supplemental Table 1 (supplemental material available online with this article; https://doi.org/10.1172/jci.insight.199050DS1).

### Sex distribution and age differences

Sex distribution was comparable across study groups, with no statistically significant differences detected (*P* > 0.05). Male participants constituted 48% of the T1D cohort, 47% of the LADA cohort, and 50% of the healthy control group. The balanced sex ratio within the control group minimized potential confounding effects in subsequent intergroup comparisons.

In contrast, a statistically significant difference in age was observed between the groups (*P* < 0.001). As anticipated, individuals with T1D exhibited a lower median age of 26 years (IQR, 21–31 years), consistent with the typical onset of autoimmune diabetes in early adulthood. The LADA group showed a significantly higher median age of 40 years (IQR, 34–45 years), reflecting the characteristic later onset of this subtype. The healthy control group had a median age of 33.5 years (IQR, 27–42 years), which was intermediate and broadly comparable to both patient groups, thereby providing an appropriate reference population for age-adjusted analyses.

### BMI and glycemic control

BMI values fell within the normal reference range across all study groups and did not differ significantly (*P* = 0.214), indicating the absence of obesity- or underweight-related confounders in metabolic comparisons.

In contrast, glycated hemoglobin (HbA1c) levels, reflecting long-term glycemic control, varied substantially among groups (*P* < 0.001). The T1D cohort exhibited the highest median HbA1c value of 7.8% (IQR, 6.4%–10.4%), consistent with the pronounced hyperglycemia characteristic of early-stage autoimmune diabetes. Participants with LADA demonstrated a moderately elevated median HbA1c of 7.1% (IQR, 6.6%–8.8%), aligning with prior observations of more gradual β cell decline in this subtype. As expected, the healthy control group maintained normoglycemic values, with a median HbA1c of 5.1% (IQR, 4.9%–5.4%).

### Higher-resolution cell composition analysis

#### Compositional analysis reveals no significant differences in cell-type proportions among disease groups.

**Figure 1:**
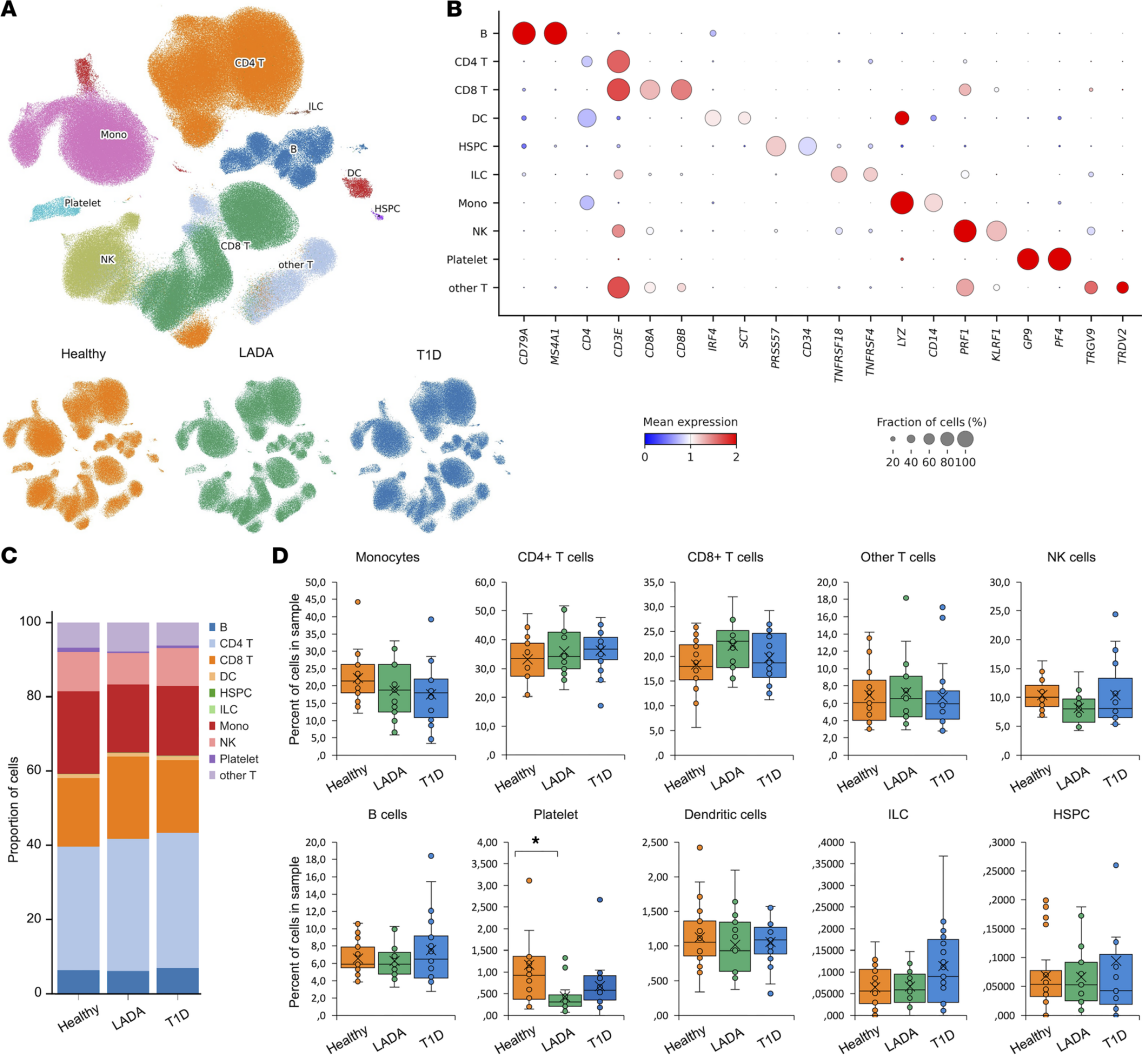
Atlas of PBMCs from healthy donors and patients with latent autoimmune diabetes mellitus and type 1 diabetes. (**A**) Uniform manifold approximation and projection (UMAP) of transcriptional profiles of PBMCs (*n* = 442,655 cells). Each cell type cluster was shown in a different color. Healthy, healthy individuals; LADA, patients with latent autoimmune diabetes mellitus; T1D, patients with type 1 diabetes. (**B**) Average expression of known marker genes in indicated cell types. The dot size represents percentage of cells expressing the genes in each cell type. The expression intensity of markers is shown. (**C**) Proportion of major cell types shown in bar plots in different donor states (Healthy, LADA, T1D). (**D**) Compositional analysis of major cell types in healthy individuals and patients with LADA and T1D. One-way ANOVA with post hoc Dunn’s test. Median (line); *n* = 15–22 (dots); *P < 0.05.

We analyzed 442,655 single-cell transcriptomes derived from PBMCs of individuals acting as healthy controls (*n* = 22), patients with LADA (*n* = 15), and individuals with newly diagnosed T1D (*n* = 21). Unsupervised clustering and uniform manifold approximation and projection identified 10 canonical immune lineages common to all 3 cohorts (Figure 1, A and B, and Supplemental Table 2). Relative frequencies of CD4^+^ and CD8^+^ T cells, B cells, NK cells, monocytes, dendritic cells, innate lymphoid cells, and hematopoietic stem/progenitor cells did not differ significantly among groups (Figure 1, C and D). One-way ANOVA followed by Dunn’s post hoc test revealed no statistically significant intergroup differences (all *P* > 0.05).

To further validate the absence of compositional skewing in peripheral immunity, we performed focused high-resolution reclustering and annotation of all T and NK cells (*n* = 316,624) across study participants (Supplemental Figure 1, A and B, and Supplemental Table 3). This analysis resolved 14 transcriptionally distinct immune subsets, corresponding to canonical T and NK cell phenotypes. Marker gene and surface protein expression profiles unambiguously delineated naive, central memory (TCM), effector memory (TEM), cytotoxic (CTL), and tissue-resident memory–like (TRM) CD4^+^ and CD8^+^ T cell subsets, along with CD56^bright^ and CD56^dim^ NK cell populations (Supplemental Figure 1, C and D).

Compositional comparisons revealed no statistically significant differences in the frequencies of any or NK cell subset among healthy donors, individuals with LADA, and patients with T1D (1-way ANOVA, Dunn’s post hoc, all *P* > 0.05; Supplemental Figure 1E). Although minor numerical trends were observed, for example, a modest elevation in CD4 TEM cells in LADA none reached significance after multiple-testing correction.

Thus, neither LADA nor T1D shows systemic leukocytosis or lineage-specific PBMC expansion, directing analyses toward qualitative perturbations in gene expression and signaling within a compositionally stable repertoire.

#### Immune signaling in LADA is attenuated compared with proinflammatory activation in T1D.

**Figure 2:**
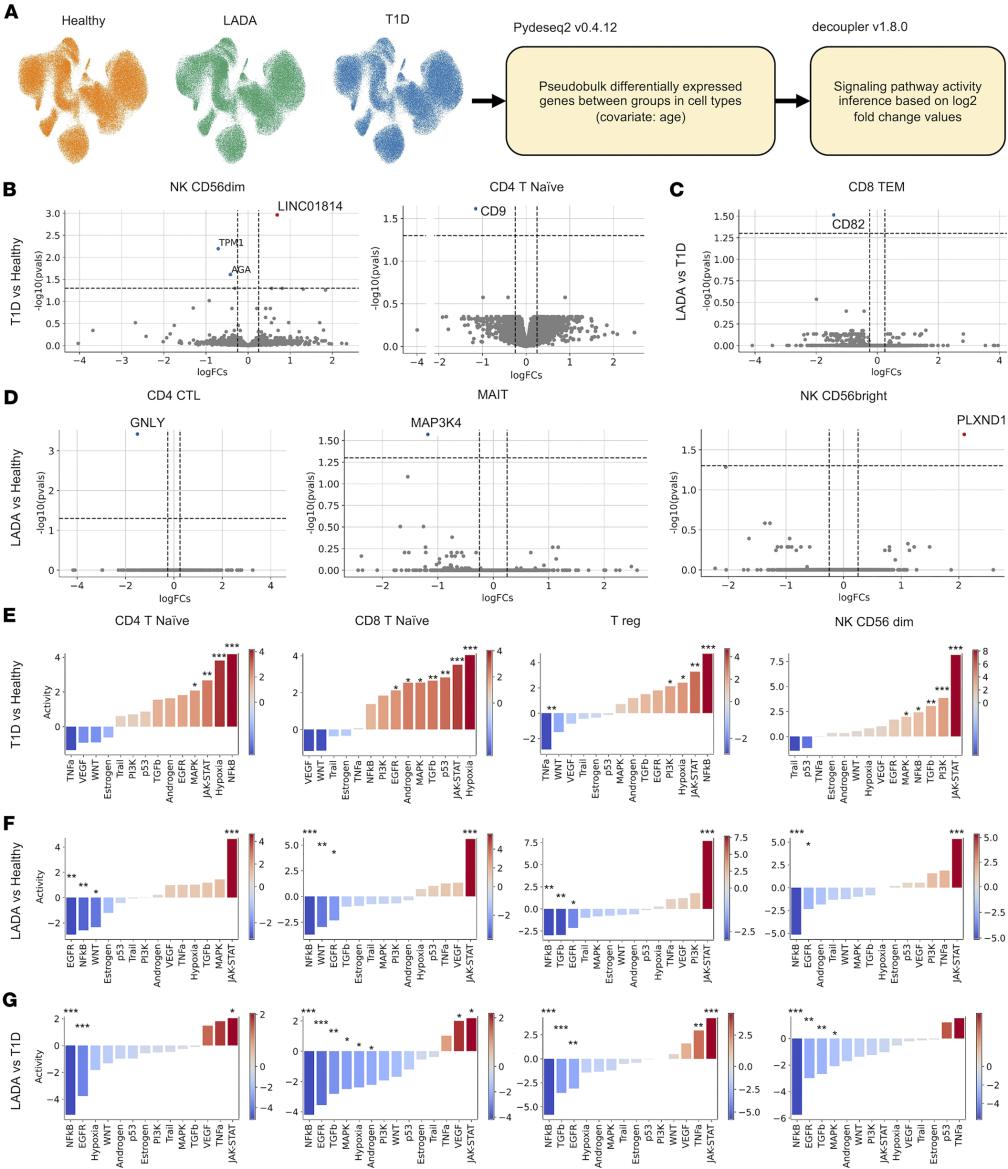
Signaling pathway activity inference in immune cells from healthy donors and patients with latent autoimmune diabetes mellitus and type 1 diabetes. (**A**) Scheme of differential gene expression analysis and pathway activity inference. (**B**–**D**) Volcano plots of differentially expressed genes in cell subtypes between patients with type 1 diabetes (T1D) and healthy donors (**B**), patients with latent autoimmune diabetes mellitus (LADA) and patients with T1D (**C**), and patients with LADA and healthy donors (**D**). Wald’s test with Benjamini-Hochberg *P* value correction. Thresholds in plots: log_2_ fold change > 0.25, *P* adjusted < 0.05. (**E**–**G**) Bar plots of differentially activated signaling pathways in cells between patients with T1D and healthy donors (**E**), patients with LADA and healthy donors (**F**), patients with LADA and patients with T1D (**G**). Student’s *t* test (2 tailed) with Benjamini-Hochberg *P* value correction. *P < 0.05, **P < 0.01, ***P < 0.001.

Differential gene expression analysis was performed using a pseudobulk approach in different T and NK cell subtypes. However, the expression changes were minor between study groups, which led us to turn to other methods of qualitative analysis (Figure 2, A–D, and Supplemental Tables 4–6). For further analysis of signaling pathway activity and cell-to-cell communications we used log_2_ fold change values of gene expression difference between study groups.

To investigate functional alterations in intracellular signaling, we quantified the activity of 14 canonical signaling cascades: androgen, epidermal growth factor receptor (EGFR), estrogen, hypoxia, JAK-STAT, MAPK, nuclear factor κ-light-chain-enhancer of activated B cells (NF-κB), tumor suppressor p53, phosphoinositide 3-kinase (PI3K), TGFB, TNFA, TRAIL, VEGF, and Wnt signaling across single-cell transcriptomes using the PROGENy algorithm (Figure 2, A and E–G, and Supplemental Tables 7–9). These pathways encompass major immunological domains, including cytokine-driven inflammation (NF-κB, JAK-STAT, MAPK), metabolic and stress response programs (hypoxia, PI3K, p53), immune regulation (TGFB, TNFA, TRAIL), and trophic or hormonal signaling (EGFR, VEGF, Wnt, androgen/estrogen).

#### Signaling pathway activity in patients with T1D compared with individuals acting as healthy controls.

In individuals with newly diagnosed T1D compared with individuals acting as healthy controls, a broad activation of inflammatory signaling was observed across T and NK cells subtypes, with consistent upregulation of NF-κB, JAK-STAT, and hypoxia-related pathways (*P* < 0.05; Figure 2E and Supplemental Table 8). Subset-specific analysis revealed that naive and central memory CD4^+^ and CD8^+^ T cells additionally exhibited elevated MAPK activity, while cytotoxic and central memory CD4^+^ T cells demonstrated increased EGFR signaling. Effector memory CD4^+^ T cells displayed a distinct gain in TGFB activity coupled with a reduction in VEGF pathway engagement.

Among innate lymphocytes, CD56^dim^ NK cells emerged as the only subset showing coordinated upregulation of PI3K and TGFB alongside the core NF-κB and JAK-STAT module. Downregulation of signaling was infrequent and largely restricted to isolated reductions in TNFA or VEGF activity, observed in select memory or innate-like subpopulations.

#### Signaling pathway activity in patients with LADA compared with individuals acting as healthy controls.

In contrast to the proinflammatory activation observed in T1D, PBMCs from individuals with LADA exhibited a broadly attenuated signaling profile across immune lineages (Figure 2F and Supplemental Table 7). Among the 14 interrogated pathways, JAK-STAT signaling was the only axis consistently retained across cell types. In contrast, NF-κB, EGFR, TGFB, MAPK, and hypoxia-related pathways were either neutral or suppressed.

In naive CD4^+^ and CD8^+^ T cells, JAK-STAT activation was preserved in the absence of accompanying inflammatory signaling, with concurrent downregulation of NF-κB and EGFR. Memory T cell and NK cell subsets exhibited broad suppression of NF-κB and EGFR activity, with occasional modest increases in TNFA or VEGF signaling that did not reach statistical significance after correction.

Notably, Tregs demonstrated the highest JAK-STAT activity within the LADA cohort, but this was accompanied by marked suppression of NF-κB, EGFR, and TGFB signaling, suggesting a unique regulatory phenotype distinct from both healthy controls and individuals with T1D.

#### Signaling pathway activity in LADA compared with T1D.

T1D is defined by a pan-cellular NF-κB/JAK-STAT/hypoxia core augmented by subset-specific protrophic signals (MAPK, EGFR, PI3K, TGFB). In contrast, LADA retains only focal JAK-STAT or sporadic TNFA/VEGF activity and broadly silences NF-κB–driven inflammation and growth factor pathways. These quantitative differences provide the molecular framework for the opposing clinical trajectories.

Direct comparison of peripheral immune cells from individuals with LADA and those with newly diagnosed T1D revealed widespread attenuation of signaling pathway activity in LADA (Figure 2G and Supplemental Table 9). Across nearly all immune subsets, nuclear factor NF-κB and EGFR signaling were significantly reduced (adjusted *P* < 0.05). Additional suppression of hypoxia-related and TGFB signaling was observed specifically in TEM cell subsets.

Residual pathway activity in LADA was largely restricted to modest preservation of JAK-STAT signaling, primarily in naive CD4^+^ and CD8^+^ T cells and Tregs. NK cells displayed only a minor upregulation of TRAIL signaling in CD56^bright^ cells, accompanied by concurrent suppression of the NF-κB, EGFR, and hypoxia pathways. Cytotoxic CD8^+^ T cells, including CTLs and TCM and TEM subsets as well as γδ T cells, exhibited pronounced downregulation of JAK-STAT activity.

Collectively, these findings highlight a striking contrast between disease states: T1D is characterized by a broad, cell-wide activation of NF-κB, JAK-STAT, and hypoxia signaling, further reinforced by subset-specific gains in growth- and survival-related pathways, such as MAPK, EGFR, PI3K, and TGFB. In contrast, LADA exhibits focal retention of JAK-STAT activity and sporadic TNFA or VEGF signaling, while broadly suppressing NF-κB–driven inflammation and trophic signaling. These quantitative differences in immune signaling architecture may underlie the divergent clinical trajectories of rapid β cell destruction in T1D versus the slower progression observed in LADA.

#### Immune receptor profiling reveals no disease-specific clonotypes among autoimmune diabetes subtypes.

**Figure 3:**
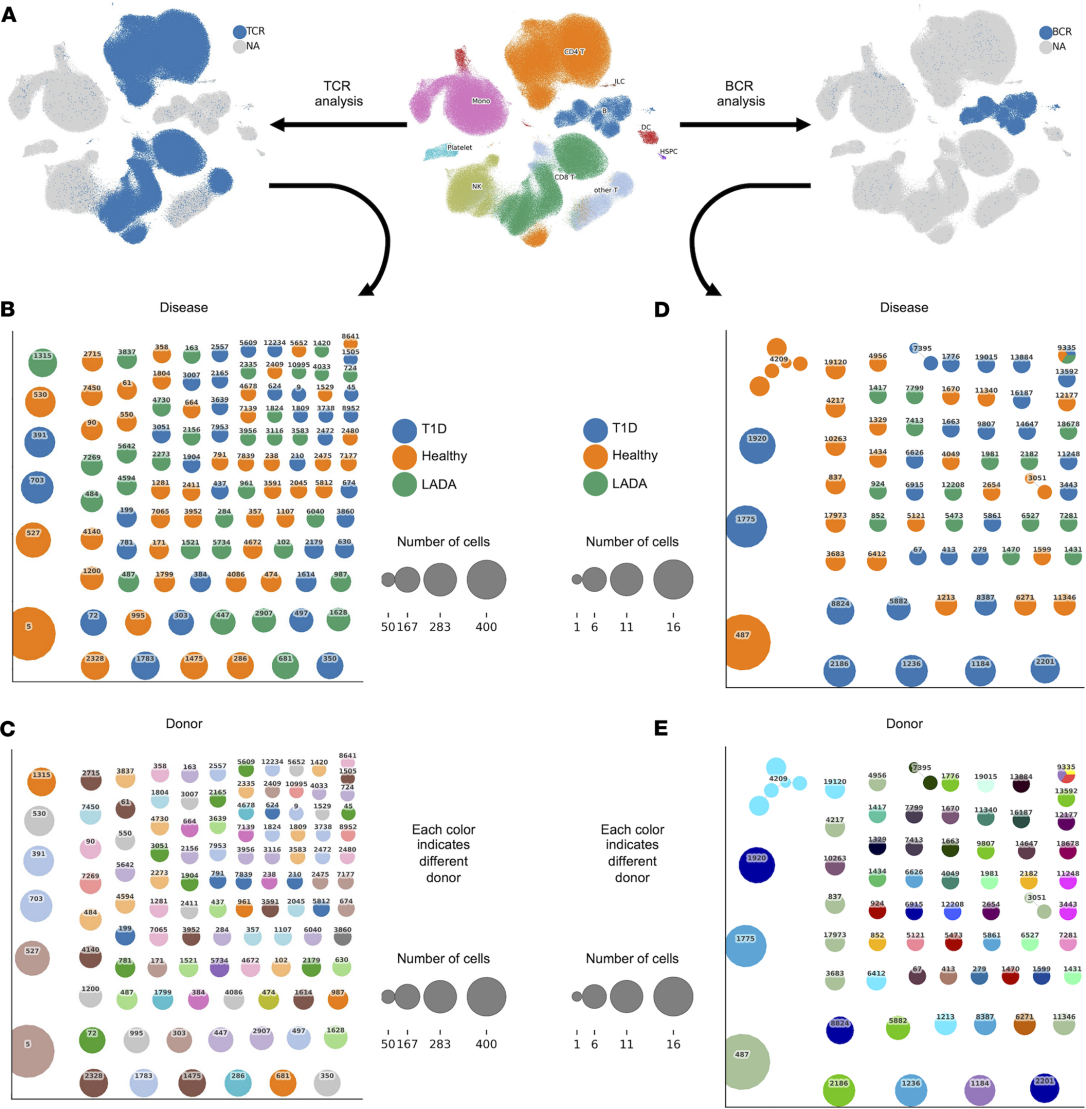
Immune receptor profiling of T and B cells from healthy donors and patients with latent autoimmune diabetes mellitus and type 1 diabetes. (**A**) Quality control of T cell receptor and B cell receptor data. (**B**) Disease-specific T cell receptor clonotype network. Each dot represents a different clone. Each color indicates a different condition: healthy donors (orange), patients with latent autoimmune diabetes mellitus (LADA, green), and patients with type 1 diabetes (T1D, blue). (**C**) Donor-specific T cell receptor clonotype network. Each dot represents a different clone. Each color indicates a different donor. (**D**) Disease-specific B cell receptor clonotype network. Each dot represents a different clone. Each color indicates a different condition: healthy donors (orange), patients with LADA (green), and patients with T1D (blue). (**E**) Donor-specific B cell receptor clonotype network. Each dot represents a different clone. Each color indicates a different donor. Grouped clones share at least 80% sequence similarity.

To dissect the specific immunological responses across different subtypes of autoimmune diabetes, we integrated scRNA-seq, T cell receptor (TCR), and B cell receptor (BCR) profiling in peripheral blood immune cells from patients with T1D, patients with LADA and individuals acting as healthy controls (Figure 3A). For TCR analysis, only cells that passed scRNA-seq quality control were included, resulting in a subset highlighted in blue (Figure 3A). We focused on T cells with available TCR information to investigate clonotype distributions. We found that both healthy donors and patients with different autoimmune diabetes subtypes exhibited entirely distinct expanded T cell clones (Figure 3, B and C). These data do not support the presence of specific clonotypes associated with the development of different autoimmune diabetes subtypes. A similar approach was used for BCR analysis (Figure 3A), focusing on B cells with available BCR information to examine clonotype distributions. We observed a comparable pattern of clonal expansion among B cells across all donor groups: each donor possessed expanded B cell clones with unique BCRs (Figure 3, D and E).

These findings indicate that the TCR and BCR repertoires in autoimmune diabetes subtypes and healthy donors are entirely private, multiclonal, and nonoverlapping, with no evidence of shared or convergent clonotypes across individuals or disease groups.

#### PD-1 expression is broadly comparable between LADA and T1D.

We evaluated PD-1 (*PDCD1*) expression across T cell subsets in patients with LADA, patients with T1D, and individuals acting as healthy controls using log_2_ fold changes; none of the contrasts reached statistical significance, so all trends should be interpreted cautiously. Focusing on LADA versus T1D, there was no robust differential PD-1 expression in CD4^+^ or CD8^+^ lineages: the CD4^+^ naive lineage showed a modest negative trend (–0.45), while CD4^+^ TCM (0.33) and CD4^+^ TEM cell lineages (0.18) trended upward; the CD8^+^ TCM cell lineage was near null (0.09), and the CD8^+^ TEM cell lineage showed a small positive shift (0.31), collectively supporting the view that PD-1 levels are broadly comparable between LADA and T1D in these compartments. Among other T cell populations, the largest nonsignificant elevation is observed in γδ T (log_2_ fold change = 1.39 in LADA vs T1D), with moderate positive trends in Tregs (0.36), mucosal-associated invariant T (MAIT) cells (0.34), and CD4^+^ CTL cells (0.42); although not statistically supported, the γδ T signal could reflect heightened activation or chronic antigenic stimulation and suggests a possible role for γδ T cell regulatory/exhaustion programs in LADA pathogenesis that warrants protein-level and functional validation.

#### Quantitative mapping of cell-cell signaling reveals amplification of pathogenic immune circuits in T1D and immune balance in LADA.

To elucidate how peripheral immune cells coordinate pathogenic signaling in autoimmune diabetes mellitus, we investigated intercellular communication patterns among T cell and innate-like lymphocyte populations. Aberrant cell-to-cell interactions are critical mediators of autoimmune tissue damage, facilitating chronic inflammation, recruitment of cytotoxic effectors, and disruption of immunoregulatory feedback loops. In the context of LADA and T1D, dysregulated ligand-receptor signaling may drive the transition from physiological immune surveillance to sustained β cell–directed autoimmunity. Single-cell resolution mapping of these communication networks enables identification of functionally distinct axes: proinflammatory, adhesive, or inhibitory.

Ligand-receptor inference was performed across the same peripheral immune subsets previously used for intracellular pathway analysis, encompassing the full spectrum of CD4^+^ and CD8^+^ T cell states, regulatory populations, and innate-like effectors. Interaction counts between T and NK cell subtypes were comparable across donor groups (Figure 3A and Supplemental Figure 2), indicating that disease-specific rewiring reflects the quality rather than the quantity of contacts. Accordingly, subsequent analyses concentrated on quantitative differences in interaction strength and on the specific ligand-receptor pairs that distinguish LADA from T1D (see also Supplemental Figures 3–8).

Peripheral immune landscape in T1D is shaped by discrete, yet interlocking, ligand-receptor circuits that jointly reinforce β cell–directed autoimmunity. Seven functional modules dominate this network: (a) a TNF-centered inflammatory and costimulatory axis; (b) enhanced antigen processing and MHC-II trafficking; (c) cytoskeletal phosphatase–mediated fine-tuning of TCR signaling; (d) global attenuation of adhesion and tissue-homing checkpoints; (e) metabolic reprogramming through basigin-SLC16A7–dependent lactate export; (f) erosion of NK cell inhibitory checkpoints driven by diminished β2-microglobulin delivery; and (g) downmodulation of noncanonical CD40L interactions. The description below details the directionality of each interaction (gain or loss of signal), the predominant cellular sources and recipients, and the inferred mechanistic consequences for T cell, NK cell, and myeloid function in the diabetic milieu. By contrast, LADA lacks these circuit amplifications: reinforcement of HLA-C–KIR and LAG3 checkpoints, together with preserved adhesion and metabolic axes, sustains a calibrated low-grade inflammatory state that underpins the immune balance described above.

#### Cell-to-cell communication — patients with T1D versus individuals acting as healthy controls.

In T1D, the peripheral immune landscape is shaped by a set of discrete yet interdependent ligand-receptor signaling modules that collectively reinforce chronic β cell–directed autoimmunity. Comparative analysis with individuals acting as healthy controls revealed 7 dominant functional clusters that define this aberrant intercellular network, described above.

These modules highlight converging mechanisms of effector amplification, antigenic persistence, and regulatory failure in the peripheral immune milieu of T1D. A comprehensive summary of differentially regulated ligand-receptor interactions, including directionality (gain or loss of signal), predominant source and target subsets, and putative functional consequences is provided in Table 1.

Collectively, the 7 modules depict a rewired PBMC network that amplifies TNF-driven inflammation, broadens the autoantigenic repertoire, lowers T cell activation thresholds, and weakens metabolic and inhibitory control; these hubs nominate TNFRSF1B, SORL1/CD74 trafficking, and KLRC1/CD94 as tractable targets to restore immune balance.

Metabolic inflexibility, marked by suppressed basigin-SLC16A7–mediated lactate export, and erosion of NK cell checkpoint control via diminished β2-microglobulin–KLRC1/KLRD1 (B2M-KLRC1/KLRD1) signaling further compound the loss of immunoregulatory restraint. Downregulation of noncanonical CD40 ligand interactions implies additional defects in antigen-presenting cell–T cell (APC–T cell) cross-activation.

By mapping these discrete but converging interaction hubs, our analysis identifies mechanistically distinct and potentially actionable nodes, such as TNFRSF1B signaling, SORL1/CD74 antigen trafficking, and KLRC1/CD94 checkpoint circuits, that may serve as immunomodulatory targets for preserving immune tolerance and attenuating β cell autoimmunity in T1D.

#### Cell-to-cell communication: patients with LADA versus individuals acting as healthy controls.

LADA exhibits a characteristically slow disease progression, which is paralleled by nuanced yet mechanistically coherent alterations in peripheral immune cell communication. Single-cell–resolved ligand-receptor inference revealed 5 functionally distinct signaling modules that differentiate individuals with LADA from individuals acting as healthy controls. (a) The first of these is reinforcement of MHC class I–KIR inhibitory checkpoints, primarily involving HLA-C and HLA-F ligands engaging KIR2DL3 and KIR3DL1 receptors on CD56^dim^ NK cells, a process consistent with NK cell education and elevated activation thresholds. (b) Stabilization of MHC class I–CD8 coreceptor interactions, through increased engagement of classical (HLA-C) and nonclassical (HLA-F) ligands with CD8A/CD8B, may reinforce the TCR-peptide-MHC interface. This may lower the activation threshold of autoreactive cytotoxic T cells and promote a persistent but nonfulminant effector state aligned with the indolent course of LADA. (c) Upregulation of MHC class II–LAG3 signaling introduces a negative feedback axis that modulates CD4^+^ T cell activity through coordinated antigen presentation and inhibitory coreceptor engagement. (d) Augmented CLEC2D-KLRB1 (LLT1-CD161) interactions support the maintenance of tissue-resident mucosal-associated invariant T (MAIT) and γδ T cell subsets, which may contribute to tissue surveillance and immune modulation. (e) Downmodulation of CD55-ADGRE5 and vimentin-CD44 axes suggests dampened complement regulatory signaling and reduced cell-matrix adhesion, potentially limiting aggressive infiltration and facilitating low-grade inflammation.

Together, these intercellular circuits define a unique immune equilibrium in LADA - one that permits the persistence of autoreactive lymphocytes while simultaneously imposing regulatory constraints that curb fulminant β cell destruction. This communication profile aligns with the indolent clinical trajectory of LADA and underscores the potential of targeting stabilizing checkpoints rather than broadly suppressing immunity as a strategy to preserve β cell function (Table 2).

Taken collectively, the LADA cell-to-cell interactome depicts a finely calibrated equilibrium in which inhibitory KIR and LAG3 pathways counterbalance reinforced MHC-I and MHC-II antigen presentation, while innate-like CLEC2D-KLRB1 signaling and attenuated adhesion/complement checkpoints sustain low-grade inflammation. This composite wiring raises activation thresholds for uncontrolled β cell destruction yet maintains sufficient effector tone to drive slow β cell attrition over time. By pinpointing nodal interactions, particularly KIR2DL3/HLA-C, CD8AB/HLA-C, and CLEC2D/KLRB1, the present analysis delineates therapeutic entry points for selective immune recalibration aimed at preserving residual β cell function in LADA.

#### Cell-to-cell communication: LADA versus T1D.

Direct single-cell ligand-receptor mapping contrasting LADA with T1D reveals 6 mechanistic modules that collectively decelerate but do not extinguish β cell–directed autoimmunity in LADA. These intercellular circuits configure a tightly calibrated immune network that stands in stark contrast to the broadly proinflammatory and cytolytic architecture characteristic of T1D. (a) Augmented MHC class I–KIR inhibitory signaling, primarily via HLA-C ligation of KIR2DL3 and, to a lesser extent, KIR3DL1, elevates the activation threshold of CD56^bright^ NK cells. This enhancement of NK cell “education” may suppress nonspecific β cell cytotoxicity and preserve immune restraint. (b) Reinforcement of MHC class I–CD8 coreceptor binding, through upregulated HLA-C and CD8A/B interactions, is hypothesized to enhance coreceptor recruitment of LCK (lymphocyte-specific protein tyrosine kinase), thereby stabilizing TCR–peptide–MHC complexes. This may sustain cytotoxic T cell activity at a sublytic threshold compatible with slow β cell attrition. (c) Lymphotoxin-β–TNFR1 (LTB-TNFR1) signaling, selectively upregulated in CD56^bright^ NK cells, may fine-tune cytokine release profiles, such as IFN-γ and TNF superfamily members, without triggering the full proinflammatory cascade observed in T1D. (d) Enhanced LLT1-CD161 (CLEC2D-KLRB1) interactions, prominently involving MAIT and γδ T cell subsets, support the maintenance of tissue-resident effector populations that propagate controlled, IL-17/IFN-γ–mediated inflammatory responses without precipitating aggressive cytolysis. (e) Neuroimmune modulation via adrenergic and ion-channel signaling, including upregulation of ARPC5-ADRB2 and CALM3-KCNQ5 ligand-receptor axes, suggests sympathetic nervous system input into effector-cell calibration. While mechanistically plausible, these interactions remain putative and require functional validation. (f) Partial erosion of the complement-adhesion checkpoint, marked by downregulation of CD55-ADGRE5 signaling, may limit deep tissue infiltration of lymphocytes but simultaneously delay the resolution of inflammation, contributing to the low-grade, smoldering immune tone characteristic of LADA.

Together, these intercellular mechanisms shape a peripheral immune architecture in LADA that allows for persistent, autoreactive immune activity while preventing the abrupt β cell destruction typical of T1D. This equilibrium may offer a therapeutic window for selective immune recalibration that preserves β cell function without impairing global immune competence (Table 3).

The LADA interactome thus represents a finely balanced equilibrium in which strengthened NK cell checkpoints counterpoise a modestly lowered CD8 activation threshold; regulatory TNF-superfamily and neuroimmune cues dampen overt Th1 polarization; and attenuated complement-adhesion safeguards sustain low-grade, chronic inflammation. This wiring pattern slows β cell attrition while preserving sufficient effector tone to drive gradual dysfunction, mechanistically explaining LADA’s protracted clinical course. Therapeutically, well-supported interaction nodes HLA-C/KIR2DL3, the LCK-linked CD8A/B complex, CLEC2D/KLRB1, and CD55/CD97 stand out as plausible targets for precision immunomodulation. In contrast, other pairs such as HLA-C–KIR3DL1, ARPC5-ADRB2, and CALM3-KCNQ5 remain preliminary and should undergo focused biochemical validation before being considered for clinical development.

#### Multiplex serum cytokine analysis.

Orthogonal serum profiling corroborated the single-cell inferences. Compared with patients with LADA, patients with new-onset T1D exhibited higher concentrations of sCD40L (16,823 pg/mL [range, 14,067–19,766 pg/mL] vs. 9,754 pg/mL [range, 6,460–13,179 pg/mL]), IL-12p70 (53.4 pg/mL [range, 41.1–159.9 pg/mL] vs. 27.5 pg/mL [range, 7.9–56.3 pg/mL]), RANTES (3,336 pg/mL [range, 2,561–3,589 pg/mL] vs. 2,467 pg/mL [range, 2,224–2,961 pg/mL]), FGF2 (523 pg/mL [range, 305–868 pg/mL] vs. 256 pg/mL [range, 152–610 pg/mL]), and IL-1α (3.82 pg/mL [range, 2.73–5.84] vs. 2.5 pg/mL [range, 1.63–2.87] pg/mL; all *P* < 0.05 within the prespecified subset). These elevations are consistent with an enhanced CD40 costimulatory axis, heightened Th1 polarization, proinflammatory chemokine signaling, and growth factor–linked tissue remodeling in T1D, collectively reinforcing the functional relevance of the signaling gradients identified at single-cell resolution.

## Discussion

### Cell composition

Single-cell profiling revealed no significant differences in peripheral proportions of CD4^+^ and CD8^+^ T, B, NK, dendritic cells, or monocytes among patients with LADA, patients with new-onset T1D, and individuals acting as controls, indicating a conserved bulk immune landscape. This contrasts with earlier flow-cytometric reports of higher effector-memory CD8^+^ T cells, altered B cell subsets, and increased activated NK cells in LADA versus T1D (4, 12, 13). Our scRNA-seq data therefore suggest that such shifts, if present, are modest and do not yield overt compositional skewing.

The key factor is individual heterogeneity: each patient shows a unique transcriptomic profile. Even after matched cohort selection and batch correction, diagnosis explained minimal variance, with no significant compositional shifts (14).

### Immune pathways driving β cell destruction

Single-cell profiling exposes a clear signaling dichotomy between LADA and new-onset T1D. In LADA, NF-κB– and EGFR-associated activities are broadly dampened, whereas T1D displays their full activation; JAK-STAT signaling is elevated in both diseases but peaks in T1D. These patterns indicate fundamentally different inflammatory set points: T1D operates in a high rate, proliferative milieu, whereas LADA maintains a muted, “dialed-down” immune tone.

#### NF-κB.

NF-κB drives proinflammatory gene expression and β cell apoptosis in T1D (15, 16). Our data show robust NF-κB activation in T1D, with attenuated NF-κB signaling in LADA, likely limiting cytokine storms and APC activation (17).

#### EGFR.

EGFR signaling, linked to cell growth and tissue repair, is likewise heightened in T1D yet minimal in LADA. In β cells, EGFR is required for compensatory proliferation after injury (18, 19); its upregulation in T1D may reflect both vigorous immune cell expansion and an unsuccessful regenerative response. Tregs can supply the EGFR ligand amphiregulin during murine insulitis (20), suggesting that the pronounced EGFR signature in T1D mirrors an acutely remodeling islet niche. The near-baseline EGFR activity in LADA implies less tissue damage and a correspondingly restrained repair program.

#### JAK-STAT.

The JAK-STAT axis integrates multiple cytokine cues, notably IFN-γ, to heighten MHC-I expression on β cells and intensify autoimmune targeting (21). Clinical and preclinical studies demonstrate that JAK1/2 inhibition (e.g., baricitinib, AZD1480) can preserve endogenous insulin production (22, 23). In our dataset, JAK-STAT activity forms a monotonic gradient, which was highest in the T1D group, intermediate in the LADA group, and lowest in the control group, underscoring its quantitative contribution to β cell loss and validating it as a tractable biomarker and therapeutic target.

Together, these contrasts position LADA and T1D along an inflammatory continuum, with NF-κB/EGFR suppression and moderated JAK-STAT activity in LADA versus full pathway activation in T1D.

### TCR and BCR repertoires

Single-cell V(D)J profiling confirmed highly private, multiclonal TCR and BCR landscapes across LADA, recent-onset T1D, and control groups: no shared CDR3 sequences or overrepresented V/J combinations emerged, corroborating earlier findings that islet-reactive lymphocytes seldom form public clonotypes in blood. This heterogeneity stems from 2 factors. (a) HLA diversity imposes idiosyncratic thymic selection, yielding patient-specific solutions to β cell epitopes. (b) Tissue sequestration concentrates pathogenic clones in islets and draining nodes; deep sequencing shows oligoclonal expansions there are absent or singletons in blood, representing <1 % of the peripheral repertoire. The same applies to B cells: the lone “public” IgH clone reported earlier is neither disease specific nor reproducible. Therefore, disease tempo depends less on convergent receptor usage than on qualitative cues, cytokine milieu, checkpoint balance, and metabolic state that tune activation of these private clones. Targeting shared signaling nodes (e.g., JAK-STAT, HLA-C–KIR) may therefore be more broadly effective than clonotype-specific approaches (24–28).

### Ligand-receptor analysis

Single-cell ligand-receptor mapping shows a fundamental rewiring of intercellular crosstalk along the diabetes spectrum. New-onset T1D displays an expansive, TNF-centric hub connecting T and innate cells, highlighted by TNF-TNFRSF1B and TNF-TRADD pairs, far exceeding healthy levels. This surge, together with TNF-driven costimulatory links such as TNF-ICOS, lowers T cell activation thresholds and amplifies bystander recruitment, cohering with TNF’s dominant β cell cytotoxicity in acute disease (1, 29). LADA PBMCs, by contrast, lack a comparable TNF module; macrophage-derived IL-1β predominates, curbing direct β cell damage and moderating overall inflammation. Therefore, T1D is wired for maximal proinflammatory signaling, whereas LADA retains a selectively attenuated, calibrated interactome.

Beyond cytokines, antigen-presentation circuits diverge sharply. T1D exhibits heightened antigen processing and MHC-II trafficking (e.g., LRPAP1–SORL1, COPA–CD74) (30, 31), expanding autoantigen loading, fostering epitope spreading, and sustaining broad T cell priming, hallmarks of rapid-onset disease with multiple autoantibodies. LADA shows no such upregulation, implying a narrower antigenic drive consistent with its tempered cytokine milieu.

Conversely, LADA displays enhanced HLA-C–CD8A/B engagement, stabilizing TCR-pMHC interactions and recruiting LCK, thereby allowing CD8^+^ CTLs to operate at a low, persistent “simmer” (32). These synapses likely support restrained effector activity that aligns with the slower β cell attrition and the reduced proliferative vigor of LADA autoreactive T cells (1). Thus, T1D favors maximal antigenic stimulation, whereas LADA pairs focused antigen engagement with regulatory restraint.

A defining hallmark of LADA is its reinforced inhibitory circuitry. Single-cell mapping shows greater engagement of HLA-C and HLA-F ligands with inhibitory KIR2DL3 and KIR3DL1 on NK cells, contrasting sharply with the attenuated contacts in T1D. These stronger KIR–HLA-C interactions likely reflect the superior inhibitory capacity of specific KIR/HLA allelic pairs; studies demonstrate that even closely related receptors (e.g., KIR2DL2 vs. KIR2DL3) dock HLA-C with distinct geometries and avidities, driving functional heterogeneity (33). In T1D the checkpoint erodes: reduced β2-microglobulin loading diminishes MHC-I display, triggering “missing-self” recognition and unleashing NK cytotoxicity (34). LADA’s upregulated KIR signaling instead preserves an “educated-self” state, curbing unwarranted β cell lysis, consistent with genetic data linking certain KIR/HLA combinations to T1D protection, an axis still underexplored in LADA (35, 36).

Parallel restraint operates in the T cell compartment. LADA shows heightened MHC-II engagement of the inhibitory receptor LAG-3 on CD4^+^ T cells, forming a feedback loop that limits proliferation (37); this axis remains largely silent in T1D. Collectively, enhanced KIR and LAG-3 signaling allows autoreactive lymphocytes to persist in LADA but under tighter surveillance, helping explain its slower, less destructive course compared with T1D.

Additional divergence occurs in adhesion and metabolic signaling. T1D PBMCs display broad suppression of homing interactions, diminished ITGAL/ITGAM-ICAM2 binding and weaker IL-16–KCNA3 chemoattractant signaling, suggesting impaired integrin-mediated transmigration and tissue residency (38, 39). Chronic hyperactivation may drive receptor shedding/internalization, leaving highly inflammatory cells sequestered in blood or lymphoid tissue and relying on TNF-mediated endothelial disruption rather than orderly diapedesis to reach islets (40). LADA retains these adhesion axes, supporting regulated, low-grade infiltration.

Metabolically, T1D lymphocytes downregulate the basigin-SLC16A7 lactate-export pathway, limiting glycolytic efflux. Resultant acid accumulation echoes tumor-associated T cell exhaustion and can dampen effector function despite heightened activation (41, 42). LADA lacks this defect: its immune cells operate below the metabolic redline, sustaining activity without overt burnout. Thus, T1D couples aggressive cytokine output with compromised migration and metabolic stress, whereas LADA maintains balanced trafficking and bioenergetic resilience.

Our interactome also revealed auxiliary signaling axes that diverge between diseases. T1D downmodulates CD40L-ITGAM, and CD40L-CD53 contacts noncanonical T cell/APC links independent of the classic CD40 pathway (43). Their loss suggests that T cell costimulation is funneled into a single, highly proinflammatory CD40 route or represents a compensatory brake against further activation. LADA retains these interactions, supporting more nuanced, balanced synapses.

Conversely, LADA uniquely upregulates CLEC2D-KLRB1 signaling on MAIT/γδ subsets, sustaining tissue-resident IL-17/IFN-γ production without triggering acute β cell cytolysis, whereas T1D skews toward overt Th1 cytotoxicity (44, 45). LADA also shows selective neuroimmune crosstalk in the ARPC5-ADRB2 and CALM3-KCNQ5 interactions, implicating adrenergic and ion channel inputs in fine-tuning immunity (46, 47). Collectively, these modulatory circuits add incremental restraints that help keep LADA’s autoimmune response below the destructive threshold characteristic of T1D.

Our findings were further supported by multiplex serum profiling, which revealed higher concentrations of sCD40L, IL-12p70, RANTES, FGF2, and IL-1α in T1D relative to LADA. These systemic readouts independently align with the TNF/CD40 costimulatory hub, Th1/JAK-STAT skewing, NF-κB–linked cytokine induction, and growth factor pathways inferred from our single-cell data. While circulating cytokine levels cannot fully capture islet-resident immune dynamics, their concordance with transcriptomic signatures reinforces the biological validity of the observed signaling divergence.

In summary, cell-to-cell communication networks in LADA versus T1D underscore a fundamental difference in immune balance. T1D is driven by potent amplification loops — TNF-centric inflammation, maximal antigen presentation, lowered activation thresholds, and lifted inhibitory checkpoints — creating a perfect storm for rapid β cell annihilation. LADA, on the other hand, embodies a state of immune equipoise: autoreactive cells are present and even primed, but they operate under heightened inhibitory surveillance, metabolic and migratory constraints, and alternative signaling that collectively enforce restraint. This delicate equilibrium in LADA’s immune crosstalk permits slow burn autoimmunity, often allowing patients to retain β cell function for years. From a therapeutic standpoint, mapping these divergent communication hubs points to tangible targets for intervention. In T1D, disrupting the TNF-TNFR axis or enhancing metabolic fitness of T cells might dampen the wildfire inflammation. In LADA, bolstering inhibitory pathways like KIR/HLA-C or LAG3, or reinforcing adhesion checkpoints, could further tip the scales toward protection without extinguishing the necessary immune surveillance. Notably, some ligand-receptor pairs identified here (for example, HLA-C–KIR2DL3 or CD8-HLA strengthening via LCK) coincide with known regulatory nodes in autoimmunity and are ripe for mechanistic exploration. By leveraging such insights, we can aim to recalibrate the immune network — quieting the excessive crosstalk in T1D and fortifying the self-restraint in LADA — to preserve pancreatic β cells while maintaining overall immune competence.

The pathogenesis of autoimmune diabetes unfolds across multiple biological levels, as demonstrated in previous studies. In the PBMC compartment, a proinflammatory immune bias is already apparent. CD4^+^ T-helper 1 cells (T-bet↑) secrete IFN-γ and IL-2, licensing the expansion and cytotoxic maturation of CD8^+^ T cells and NK cells, whereas Th17 cells (RORγt↑) produce IL-17A, fostering monocyte recruitment (48, 49). Concomitantly, quantitative and functional attrition of FOXP3^+^ Tregs weakens peripheral tolerance (50, 51). B cell dysregulation manifests as autoreactive clones synthesizing insulin, GAD65, IA-2, and ZnT8 antibodies, while the innate compartment, classical monocytes exhibit NF-κB and STAT1/3 activation, further amplifying systemic priming (52, 53).

Antigen-laden dendritic cells traffic via the lymphatics to pancreatic draining lymph nodes, where β cell peptides are displayed on MHC I/II, driving clonal expansion of CD8^+^ cytotoxic and CD4^+^ effector subsets together with germinal center maturation of B cells (54). The emerging repertoires are oligoclonal and individualized, lacking a universal public TCR, yet collectively they seed the circulation and home to the pancreas along (55).

Within the islets of Langerhans, infiltrating CD8^+^ CTLs and NKG2D NK cells execute granzyme-B/perforin-mediated β cell lysis, while Th1- and Th17-derived cytokines (IFN-γ, IL-17A) heighten local inflammation and numerically insufficient Tregs fail to restore restraint (56, 57). Stressed β cells characterized by HLA-I upregulation, ER perturbation, and CXCL10 release activate JAK-STAT1/3, NF-κB, PI3K-AKT-mTOR and MAPK p38/JNK pathways, culminating in apoptosis, secondary antigen liberation, and epitope spreading (58–60). Type I interferons perpetuate this feed-forward loop, establishing chronic pancreatic inflammation that feeds back into systemic immune activation and metabolic dysregulation.

**Figure 4:**
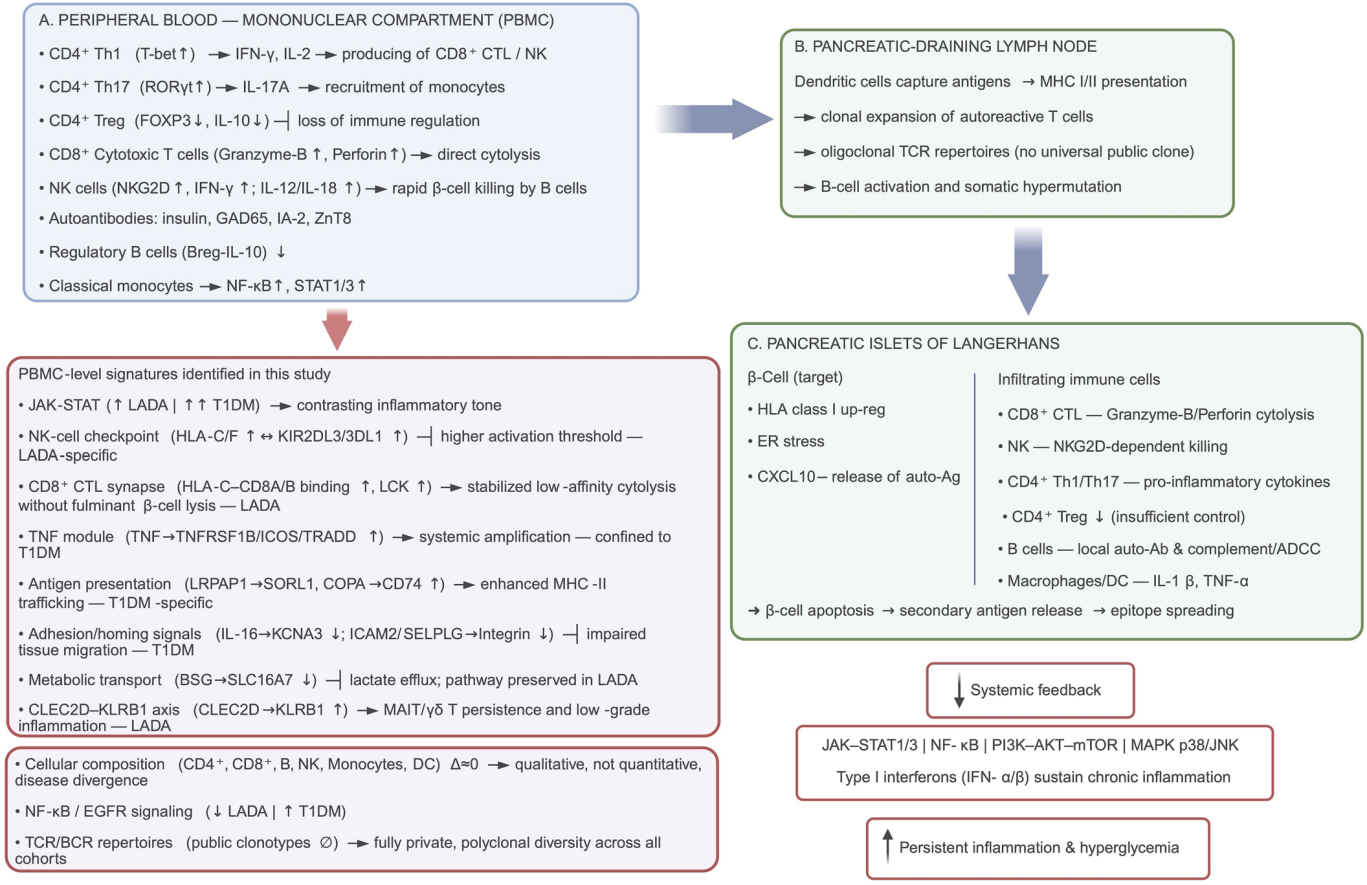
Model of T1D and LADA pathogenesis based on published literature and our own findings. (**A**) Illustration of the PBMC compartment, where heightened activity of CD4^+^ Th1/Th17, CD8^+^ cytotoxic T lymphocytes (CTLs) and NK cells, coupled with depletion of FOXP3^+^ Tregs and IL-10–producing B regulatory cells (Bregs), establishes a proinflammatory milieu and systemic production of disease-defining autoantibodies. (**B**) Depiction of the pancreatic draining lymph node: dendritic cells ferry β cell antigens, driving oligoclonal expansion of autoreactive T cell receptors (TCRs) without a universal public clonotype. (**C**) Illustration of the islet microenvironment, where upregulation of HLA class I molecules and endoplasmic reticulum stress in β cells result in their direct lysis by CD8^+^ CTLs and NK cells. In addition, β cells are subject to cytokine-mediated damage driven by Th1 and Th17 cells, antibody-dependent cellular cytotoxicity (ADCC), and the effects of innate cytokines released by macrophages and dendritic cells. The bottom left panel displays data generated in the present study. Integrated JAK-STAT1/3, NF-κB, PI3K-AKT-mTOR, MAPK p38/JNK pathways maintain chronic inflammation and propagate β cell apoptosis, leading to epitope spreading and progressive loss of insulin secretion. Created in BioRender. (Samsonova M, 2025; https://BioRender.com/tkecn1v).

As with most human diabetes studies, our analyses were confined to peripheral blood rather than islet tissue, limiting direct mechanistic inference. To synthesize the above observations, we present an updated, multitier model of autoimmune diabetes pathogenesis (Figure 4) that incorporates the key single-cell discoveries of this study. Specifically, the diagram highlights (a) the selective dampening of NF-κB– and EGFR-driven signaling cascades in LADA, contrasted with their robust activation in new-onset T1D, while JAK-STAT activity remains comparably elevated in both conditions; (b) the strengthened inhibitory checkpoint formed by HLA-C engagement of KIR2DL3/3DL1, which raises the activation threshold of NK cells in LADA; (c) reinforcement of the HLA-C–CD8A/B coreceptor complex that stabilizes low-affinity cytotoxic T cell interactions without precipitating fulminant β cell lysis; (d) augmented CLEC2D-KLRB1 signaling that sustains a tissue-resident, low-grade inflammatory tone; and (e) the absence of major shifts in peripheral immune cell composition, underscoring that qualitative, rather than quantitative, rewiring of immune circuitry distinguishes LADA from T1D. Together, these integrated elements provide a coherent framework explaining how LADA maintains chronic, yet restrained, β cell autoimmunity, whereas T1D is driven by an unchecked proinflammatory network that accelerates β cell destruction. Our data do not establish LADA as a distinct disease entity. Rather, they support the interpretation of LADA and T1D as points along an autoimmune diabetes continuum, differentiated by inflammatory set points and rate of β cell decline. Although age was modeled as a covariate, residual influences of age and disease duration cannot be fully excluded.

### Limitations and future directions

Our study has several limitations that should be acknowledged. First, it is cross-sectional and, thus, cannot establish temporal causality. Second, although age was modeled as a covariate, residual influences of age and disease duration cannot be fully excluded. Median HbA1c values were comparable between T1D and LADA (7.8% vs 7.1%), and all patients with T1D as well as 11 of 15 patients with LADA were on insulin therapy, minimizing — but not eliminating — the contribution of metabolic control and treatment status. Third, our analyses relied on PBMCs rather than islet-infiltrating cells, which may differ in situ. Fourth, we did not perform orthogonal functional assays such as immunoblotting or receptor blockade to directly test HLA-C–KIR or HLA-C–CD8 interactions. Finally, the absence of an independent replication cohort limits the generalizability of our findings. These caveats underscore the need for future longitudinal and mechanistic studies to validate and extend our observations. Nevertheless, by integrating single-cell transcriptomics with multiplex cytokine profiling, our work provides a framework that generates testable hypotheses for follow-up functional and clinical investigations.

### Conclusion

In this single-cell study, we constructed a high-resolution immune atlas of LADA and new-onset T1D, analyzing more than 4 × 10^5^ peripheral blood mononuclear cells together with paired V(D)J repertoires. Contrary to earlier flow cytometric reports, we found no quantitative shift in the frequencies of major lymphoid or myeloid lineages between the two disease entities and individuals acting as healthy controls, indicating that the pace of β cell destruction is dictated by qualitative rather than numerical immune alterations. Transcriptome-derived pathway scores revealed diametrically opposed signaling landscapes: T1D displayed broad, pan-lineage activation of NF-κB, EGFR, MAPK, and hypoxia programs, whereas LADA was marked by a global suppression of NF-κB/EGFR cascades, with selective retention of a moderate JAK-STAT tone. This molecular gradient establishes distinct inflammatory set points that are commensurate with the clinical velocity of autoimmunity.

Intercellular communication mapping further showed that LADA is enriched for inhibitory checkpoints, most notably HLAC/F engagement of KIR2DL3/3DL1 on NK cells and reinforcement of the HLAC-CD8A/B-LCK coreceptor complex, thereby raising the activation threshold of cytotoxic effectors while preserving low-affinity autoreactivity. In striking contrast, T1D is driven by an expansive TNF-centered crosstalk, augmented MHC-II trafficking and coordinated erosion of adhesion and homing signals, all of which synergistically potentiate systemic inflammation and tissue infiltration. Metabolically, basigin-mediated lactate export is preserved in LADA but suppressed in T1D, suggesting greater glycolytic stress in the latter, while the CLEC2D-KLRB1 (LLT1-CD161) axis is uniquely upregulated in LADA, sustaining tissue-resident MAIT/γδ T cell activity and low-grade inflammation.

The absence of public T or B cell clonotypes across cohorts underscores that disease trajectory is governed by the cytokine milieu and checkpoint balance rather than convergent receptor usage. Taken together, our findings refine the conceptual framework of autoimmune diabetes, positioning LADA and T1D at opposite ends of a continuum defined by the degree of inflammatory amplification versus inhibitory restraint. These findings refine, but do not resolve, the debate on whether LADA constitutes a separate disease entity or a slower trajectory within autoimmune diabetes. Clinically, the NF-κB/EGFR–JAK-STAT gradient offers a tractable molecular signature for early risk stratification, while the HLAC-KIR and HLAC-CD8A/B-LCK axes emerge as attractive targets for precision immunomodulation aimed at preserving residual β cell function.

A key limitation is that our analyses were confined to peripheral blood rather than pancreatic tissue, which may not fully reflect the local immune environment at the site of β cell destruction.

## Methods

### Sex as a biological variable.

Both male and female participants were enrolled. Sex was not used as a stratification factor, and no sex-specific analyses were performed. Findings are expected to be relevant to both sexes.

### Study design and participants.

Participants were not randomized, and group allocation was known to investigators prior to analysis, as clinical group designation was essential for stratification and subsequent comparative analyses.

This was a single-center, cross-sectional, observational study. Participants were recruited consecutively, including all eligible individuals who received either inpatient or outpatient care at the study site during the enrollment period.

This study was conducted at the Endocrinology Research Center, Ministry of Health of the Russian Federation, between February 2023 and December 2024. A total of 58 individuals were enrolled and stratified into 3 groups: individuals acting as healthy controls (*n* = 22), patients with newly diagnosed T1D (disease duration, ≤1 year, *n* = 21), and patients with LADA (disease duration, ≤5 years, *n* = 15). Detailed inclusion and exclusion criteria are summarized in Table 4.

### Clinical assessments.

Fasting plasma glucose and glycated hemoglobin (HbA1c) levels were measured using the Architect c8000 automated biochemical analyzer (Abbott Laboratories), following protocols standardized by the National Glycohemoglobin Standardization Program.

### Sample collection and processing.

The work was carried out using the materials of the Unique Scientific Facility “Collection of Biological Material from Patients with Endocrine Pathologies” of the Endocrinology Research Center.

Venous blood collected for fasting glucose and HbA1c were placed into BD Vacutainer tubes with gel separator or EDTA (4 mL). PBMCs were isolated from whole blood in BD Vacutainer CPT tubes with sodium heparin and ficoll (8 mL) and processed within 2 hours at room temperature.

PBMCs were isolated by centrifugation in a horizontal rotor (swing-out head) at 20°C for 20 minutes at 1,800 RCF. The upper 5 mL of plasma was discarded, and the remaining fraction, including the PBMC layer, was transferred to 50 mL conical tubes for washing. Cells were washed twice with 1× PBS supplemented with 1% heat-inactivated FBS (HyClone) and 1 mM EDTA, followed by centrifugation at 300 RCF for 10 minutes. Residual red blood cells were lysed using ACK Lysing Buffer (Thermo Fisher Scientific), followed by additional wash and centrifugation steps. The final PBMC pellet was resuspended in CryoStor CS10 (StemCell Technologies) and stored at –80°C (short-term) or –150°C (long-term).

### Sample preparation for scRNA-seq.

Cryopreserved PBMCs were rapidly thawed in a 37°C water bath for 3 minutes and then transferred gently into 14 mL RPMI-1640 medium (PanEco) supplemented with 10% heat-inactivated FBS (HyClone). Cells were centrifuged at 20°C for 10 minutes at 300 RCF, and the resulting pellet was resuspended and passed through a 30 μm MACS SmartStrainer (Miltenyi Biotec) to remove cell aggregates. All subsequent procedures were performed on ice to preserve cell viability and transcriptome quality.

Viable cell numbers were determined using trypan blue exclusion with the Countess 3 FL automated cell counter (Thermo Fisher Scientific). The final cell suspension was adjusted to a concentration of 3,000 cells/μL in RPMI-1640 supplemented with 10% FBS for downstream single-cell library preparation.

### scRNA-seq.

scRNA-seq was performed using the Chromium Next GEM Single Cell 5′ Reagent Kits v2 (Dual Index) according to the manufacturer’s protocol (10x Genomics). PBMC suspensions containing approximately 50,000 cells per sample were loaded into microfluidic chips and processed using the Chromium Controller for single-cell partitioning and barcoding.

Library quality was assessed using the Qubit dsDNA High Sensitivity Assay Kit (Thermo Fisher Scientific) for DNA quantification and the Agilent High Sensitivity D1000 ScreenTape Assay (Agilent Technologies) for fragment size analysis. Sequencing was performed on the NovaSeq 6000 platform (Illumina) using the NovaSeq 6000 S4 Reagent Kit v1.5 (300 cycles) in paired-end mode (151–10–10–151 cycles; 151 bp for read 1 and read 2; dual 10 bp i7 and i5 sample indices), with libraries multiplexed at concentrations of 1.3–1.4 nM for 5′ gene expression libraries (standard NovaSeq protocol) and 1–1.1 nM for V(D)J libraries (NovaSeq XP protocol). The expected sequencing depth was approximately 80,000 read pairs per cell for 5′ gene expression library and about 15,000 read pairs for V(D)J library

### Single-cell RNA-seq analysis.

Raw sequencing scRNA-seq data were processed using bcl2fastq2 12.20 to generate FASTQ files. Reads were aligned to the GRCh38-2020-A human reference genome using Cell Ranger 7.1.0. Ambient RNA contamination was removed using SoupX 1.6.2, and doublets were identified using scDblFinder 1.12.0 in R 4.2.1. High-quality cells were retained based on the following criteria: mitochondrial gene content, <6%; heat shock protein gene content, <2%; and at least 200 expressed genes per cell.

Further analyses were conducted using scanpy 1.10.2 in Python 3.9.16. Data were log-normalized, and 2,000 variable genes were identified with the seurat_v3 method. Integration was performed using scANVI. Cell-type annotation was performed with scParadise and Azimuth, supplemented by marker databases (CellMarker 2.0, PanglaoDB, GeneCards). Surface protein data imputation was performed using pretrained the Human_PBMC_3p scEve model from scParadise tool. Differential gene expression analysis between cell populations was conducted in scanpy 1.10.2 using Wilcoxon’s test. We used the following criteria to filter differentially expressed genes for generating cell-type–specific marker gene lists: log_2_ fold change, >1.0; proportion of cells within the cell type where the gene is detected, >0.2’ and Benjamini-Hochberg adjusted *P* value <0.05. Differential gene expression analysis between donors was performed using pseudobulk analysis with pyDeSeq v0.4.12. Statistical analysis of differential gene expression was conducted using the Wald’s test with Benjamini-Hochberg *P* value correction. Age was included as a covariate in all differential expression and pathway models to reduce potential confounding between LADA and T1D cohorts.

Signaling pathway activity was determined using a multivariate linear model in decoupler 1.8.0 and the PROGENy database. Signaling pathway activity inference was performed using the log_2_ fold change values from the list of differentially expressed genes. Statistical testing of signaling pathway activity was performed using Student’s *t* test (2 tailed) with Benjamini-Hochberg *P* value correction. Cell composition analysis across the study groups was conducted using pertpy 0.9.4

Ligand-receptor interaction analysis was performed using liana (v1.5.0). To enhance robustness and reduce methodological bias, we used an aggregate score that integrates results from 5 independent methods: CellPhoneDB, Connectome, log_2_ fold change, Network Analysis Toolkit for Multicellular Interaction, and SingleCellSignalR. A consensus database of ligand-receptor interactions was also used to maximize the scope of detected interactions. We used differentially expressed genes previously identified for different donor groups to reflect variations in ligand and receptor expression between diabetes subtypes. This strategy enabled the identification of communication events specific to particular groups and provided additional biological insight.

### Single-cell TCR/BCR-seq analysis.

Raw sequencing scTCR/BCR-seq data were processed using bcl2fastq2 12.20 to generate FASTQ files and Cell Ranger 7.1.0 and bcl2fastq2 12.20 to generate FASTQ files. Reads were aligned to the vdj-GRCh38-alts-ensembl-7.1.0 human reference genome using Cell Ranger 7.1.0. We performed quality control of TCR and BCR data by retaining only those cells that passed the quality control filtering during the processing of the scRNA-seq gene expression matrices. Thus, only cells with high-quality transcriptomic profiles were included in the subsequent TCR/BCR analyses. Cells were grouped into clonotypes based on the nucleotide sequence identity (for TCR data) and 80% similarity (for BCR data) of the CDR3 region. We used at least 50 cells (for TCR data) and 3 cells (for BCR data) for clonotype network visualization (61–78).

### Multiplex serum cytokine analysis.

The level of various immunoregulatory factors in the serum was measured using a commercial kit (MILLIPLEX Human Cytokine/Chemokine/Growth Factor Panel A 48 Plex Premixed Magnetic Bead Panel, HCYTA-60K-PX48, Millipore Sigma) using the MAGPIX (Luminex Corp). The analysis was performed according to the manufacturer’s instructions. xPONENT 4.1 software was used to obtain the raw data. The concentrations of 48 cytokines were determined using standard curves.

### Use of artificial intelligence.

Generative AI (GPT-4o, OpenAI) was used in August 2025 to assist with English language editing and partial translation of the manuscript from Russian to English. Prompts were limited to requests for grammatical correction, stylistic refinement, and clarification of scientific English phrasing. All scientific content, data interpretation, and conclusions were written, reviewed, and verified by the authors.

### Statistics.

Clinical and cytokine data were analyzed in Statistica (v13). Continuous variables are presented as median (IQR) and were compared using the Kruskal-Wallis test followed by 2-tailed Mann-Whitney *U* post hoc comparisons. Categorical variables were analyzed using the χ² test. A *P* value of less than 0.05 was considered statistically significant.

### Study approval.

The study was approved by the local ethics committee of the Endocrinology Research Centre (protocol no. 18, October 12, 2022). Written informed consent was obtained from all participants.

### Data availability.

ScRNA-seq data generated for this manuscript are available in the GEO database under accession number GSE308968. Supporting data values underlying all main and supplemental figures are provided in the Supporting Data Values file.

## Author contributions

IIG and MVS conceived the research concept. IIG, ESP, AVZ, and AAM developed the wet-lab methodology, carried out the experiments, and performed initial data analyses. VIC designed and executed the bioinformatic pipeline, validated the results, and, together with IIG and MDS, generated the figures. YVD, TVN, EVB, MYL, and YAM provided scientific expertise. DNL, RIK, and NAS contributed to data interpretation. IRM, MVS, and NGM acquired funding, provided project administration, and supervised the study. IIG and VIC wrote the first draft of the manuscript, and all authors critically revised, edited, and approved the final version.

## Funding support

- Ministry of Science and Higher Education of the Russian Federation (agreement no. 075-15-2024-645 from July 12, 2024).

## Supplementary Material

Supplemental data

Supplemental table 1

Supporting data values

## Figures and Tables

**Table 1:**
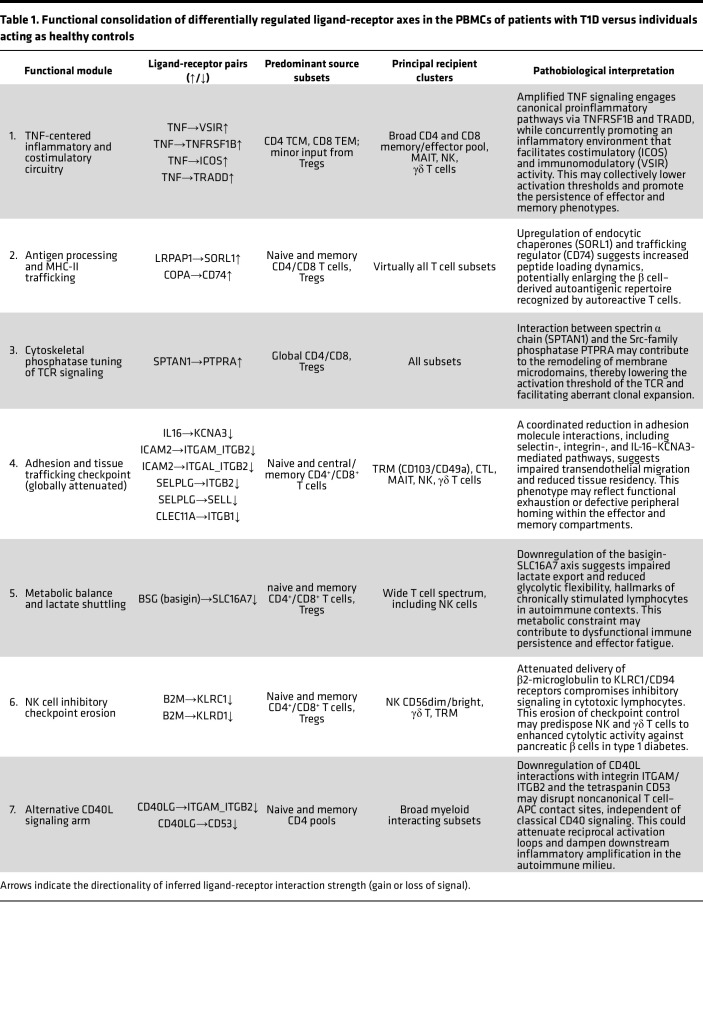
Functional consolidation of differentially regulated ligand-receptor axes in the PBMCs of patients with T1D versus individuals acting as healthy controls

**Table 2:**
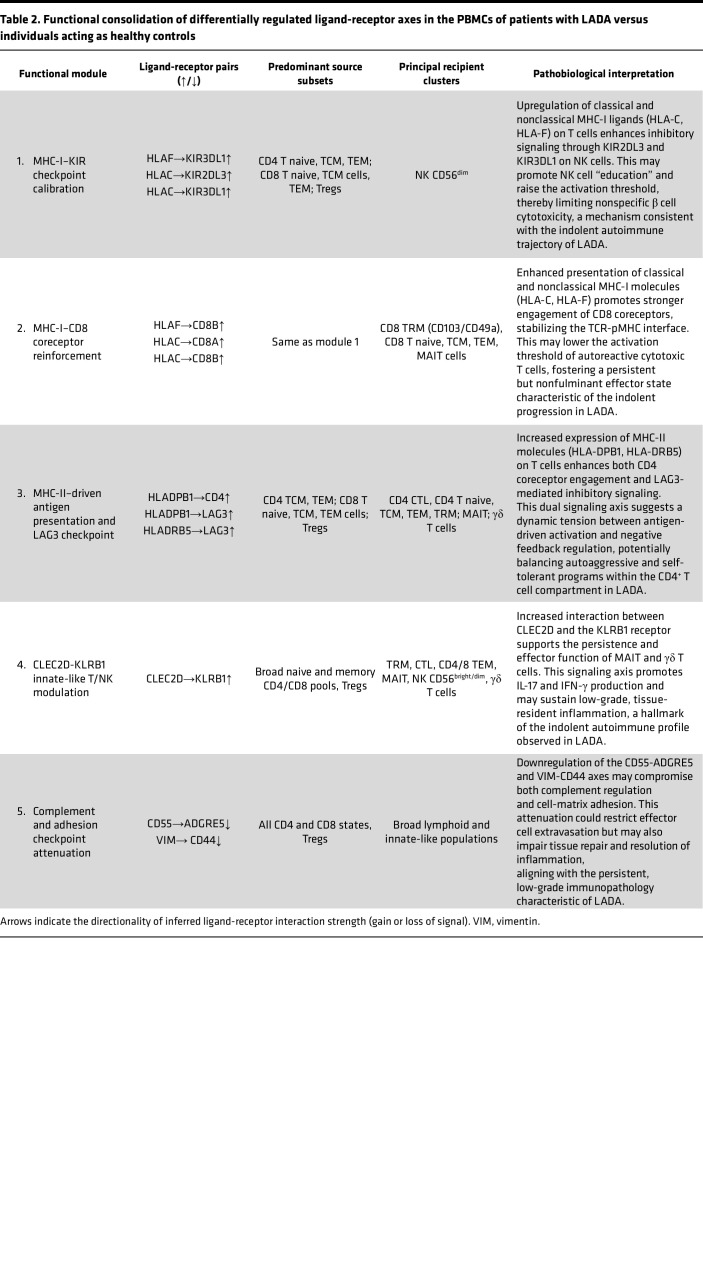
Functional consolidation of differentially regulated ligand-receptor axes in the PBMCs of patients with LADA versus individuals acting as healthy controls

**Table 3:**
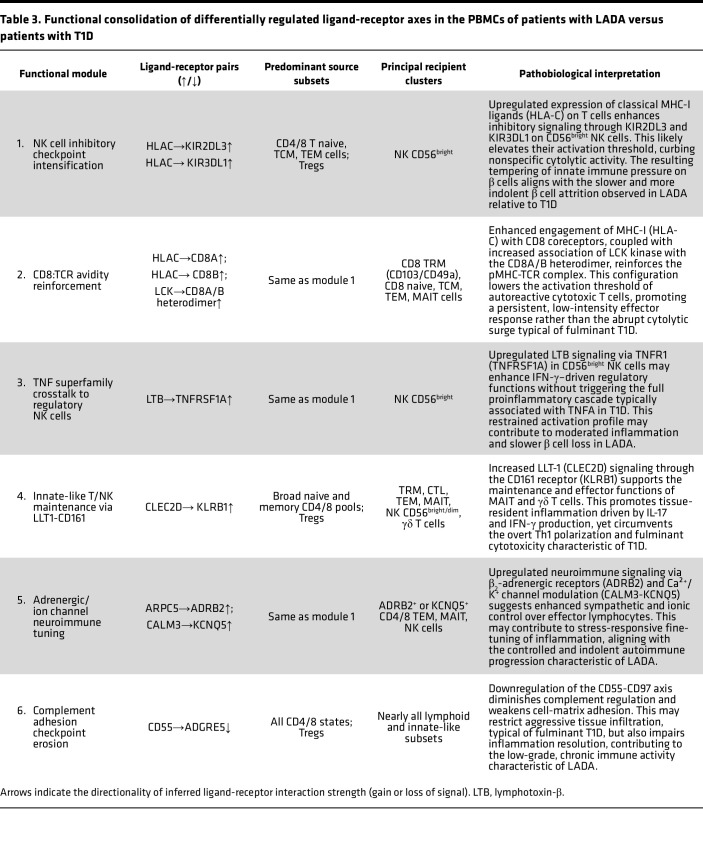
Functional consolidation of differentially regulated ligand-receptor axes in the PBMCs of patients with LADA versus patients with T1D

**Table 4:**
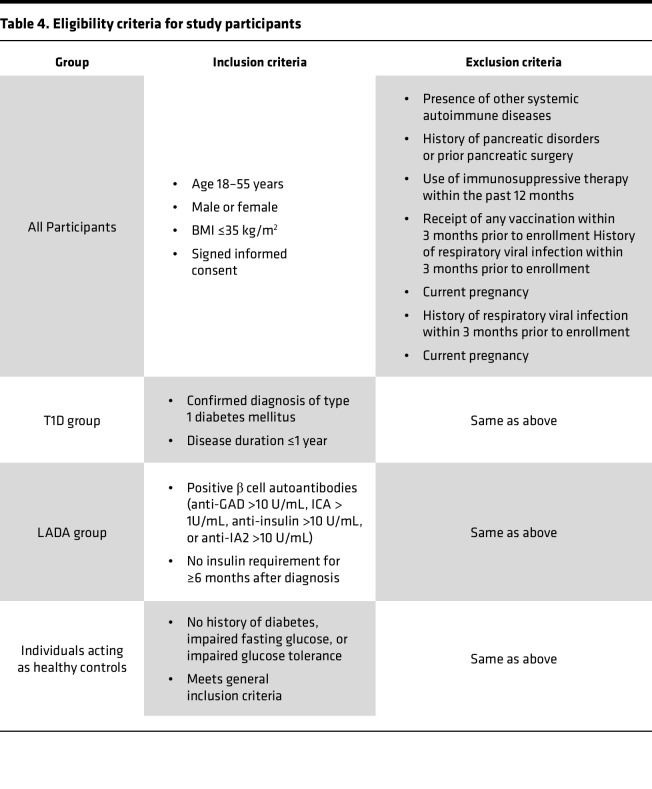
Eligibility criteria for study participants

## Version 1 12/01/2025

In-Press Preview

## Version 2 01/09/2026

Electronic publication
