# Supplementary material for: Single-cell immune transcriptomics reveals an inflammatory-inhibitory set-point spectrum in autoimmune diabetes
Source: JCI Insight. 2025 Nov 25;11(1):e199050. doi: 10.1172/jci.insight.199050 (PMC12890478; doi:10.1172/jci.insight.199050)
Supplement: Supplemental data [file jciinsight-11-199050-s096.pdf]

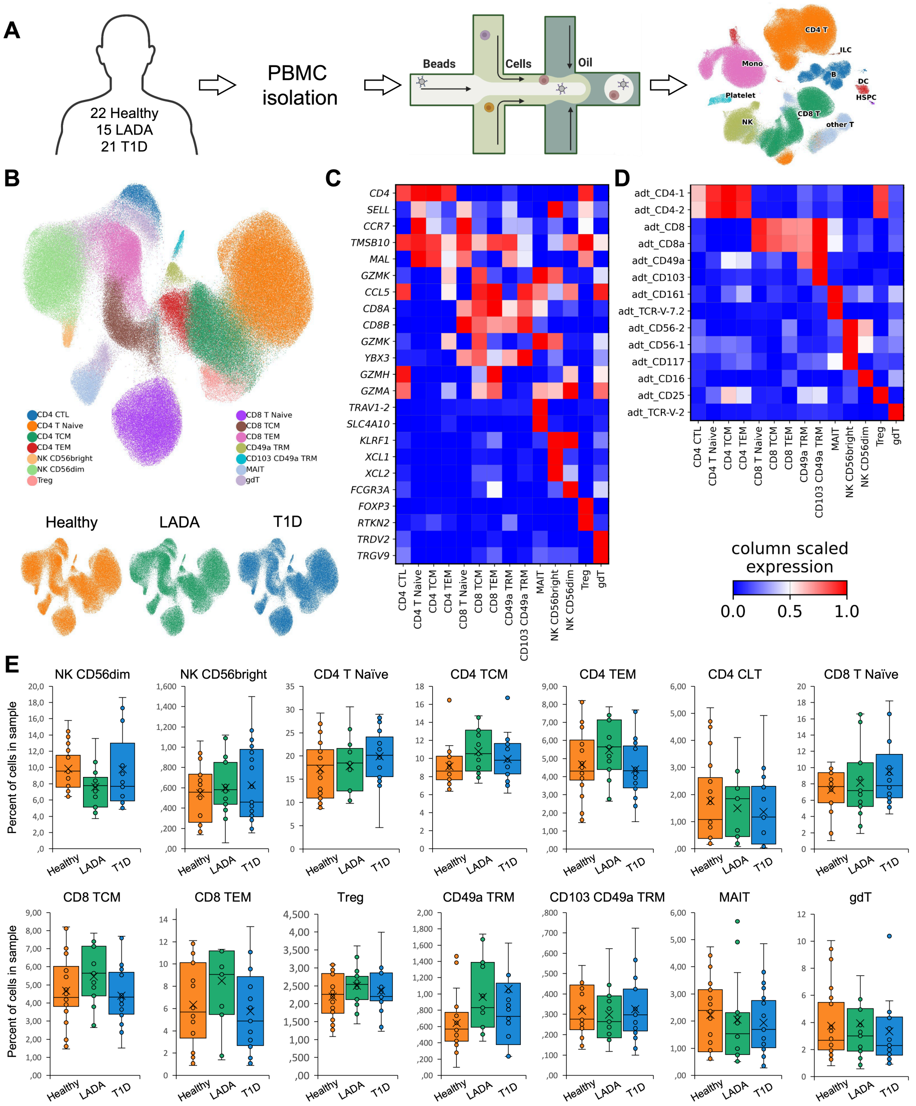

Supplementary Figure 1. Atlas of T and NK cell subtypes from healthy donors (Healthy) and patients with latent autoimmune diabetes mellitus (LADA) and type 1 diabetes (T1D). (**A**) Scheme of scRNA-seq experiment of PBMC from 22 healthy donors, 15 patients with latent autoimmune diabetes mellitus, and 21 with type 1 diabetes. (**B**) Uniform manifold approximation and projection (UMAP) of transcriptional profiles of T and NK cells (n = 316624 cells). Each cell type cluster was shown in different color. Healthy, healthy individuals; LADA, patients with latent autoimmune diabetes mellitus; T1D, patients with type 1 diabetes. (**C**) Matrix plot showing scaled expression of known marker genes in indicated cell types. (**D**) Matrix plot showing scaled expression of known marker proteins (imputed) in indicated cell types. (**E**) Compositional analysis of T and NK cell subtypes in healthy individuals and patients with latent autoimmune diabetes mellitus (LADA) and type 1 diabetes (T1D). One-way ANOVA showed no statistically significant differences between groups (Healthy, LADA, T1D). Median (line), mean (cross), n = 15-22 (dots), *p < 0.05.

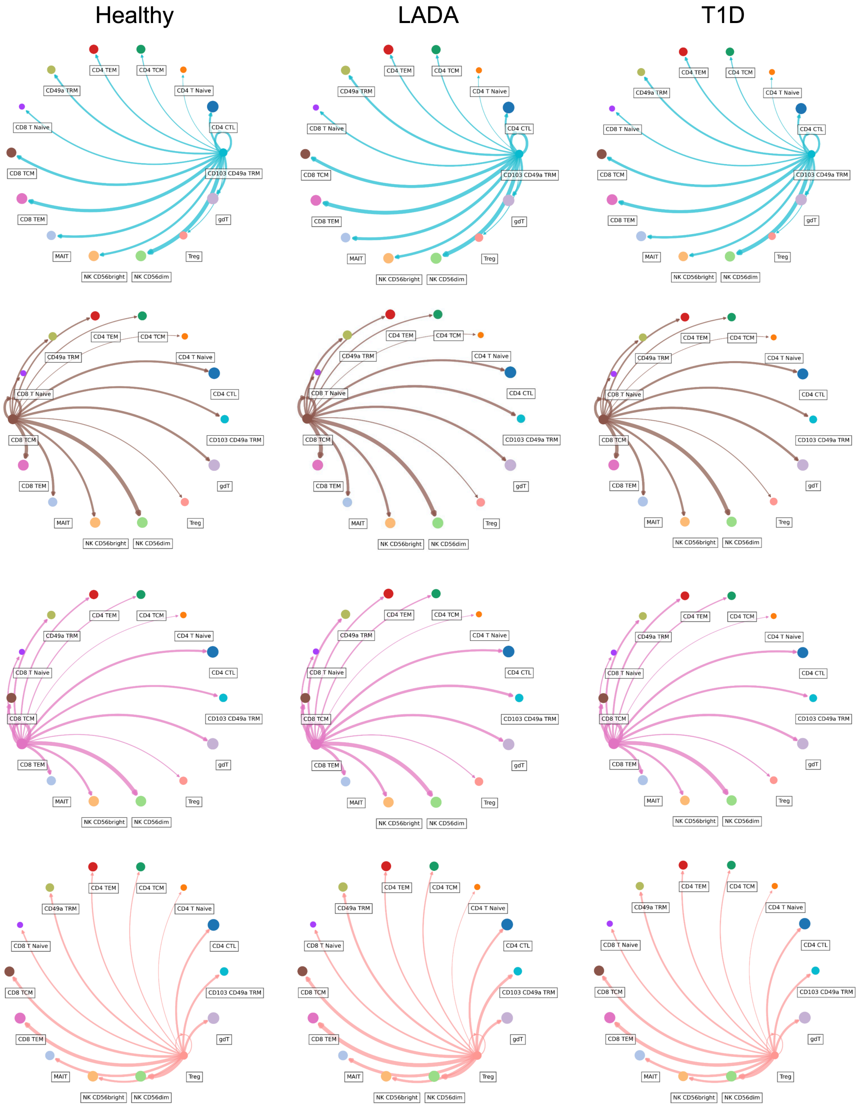

Supplementary Figure 2. Ligand–receptor interaction counts between T and NK cell subtypes. Each arrow represents the number of ligand–receptor interactions between cells. The origin of the arrow indicates the source of ligands, while the arrowhead points to the cell population expressing receptors for the secreted ligands. This figure compares number ligand-receptor interactions of CD103+ CD49a+ tissue resident T cells (TRM), CD8+ central memory T cells (TEM), CD8+ effector memory T cells (TEM) and T regulatory cells (Treg) from healthy donors (Healthy) and patients with latent autoimmune diabetes mellitus (LADA) and type 1 diabetes (T1D).

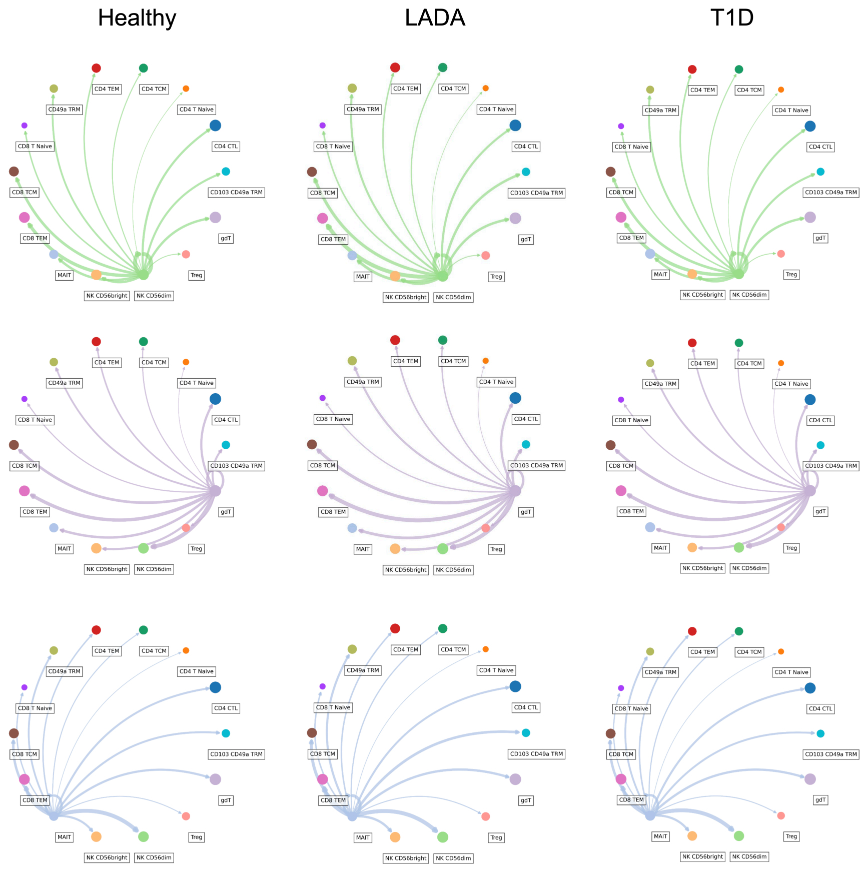

Supplementary Figure 3. Ligand–receptor interaction counts between T and NK cell subtypes. Each arrow represents the number of ligand–receptor interactions between cells. The origin of the arrow indicates the source of ligands, while the arrowhead points to the cell population expressing receptors for the secreted ligands. This figure compares number ligand-receptor interactions of NK CD56dim cells, mucosal-associated T cells (MAIT) and gamma-delta T cells (gdT) from healthy donors (Healthy) and patients with latent autoimmune diabetes mellitus (LADA) and type 1 diabetes (T1D).

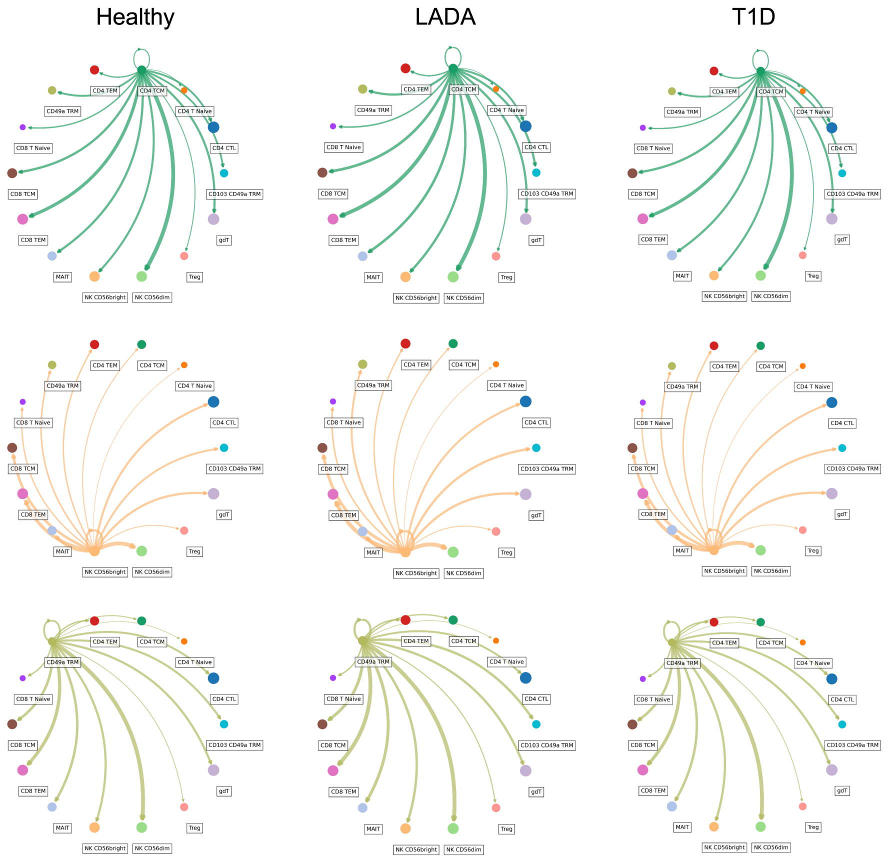

Supplementary Figure 4. Ligand–receptor interaction counts between T and NK cell subtypes. Each arrow represents the number of ligand–receptor interactions between cells. The origin of the arrow indicates the source of ligands, while the arrowhead points to the cell population expressing receptors for the secreted ligands. This figure compares number ligand-receptor interactions of CD4+ central memory T cells (TCM), NK CD56bright cells and CD49a+ tissue resident T cells (TRM) from healthy donors (Healthy) and patients with latent autoimmune diabetes mellitus (LADA) and type 1 diabetes (T1D).

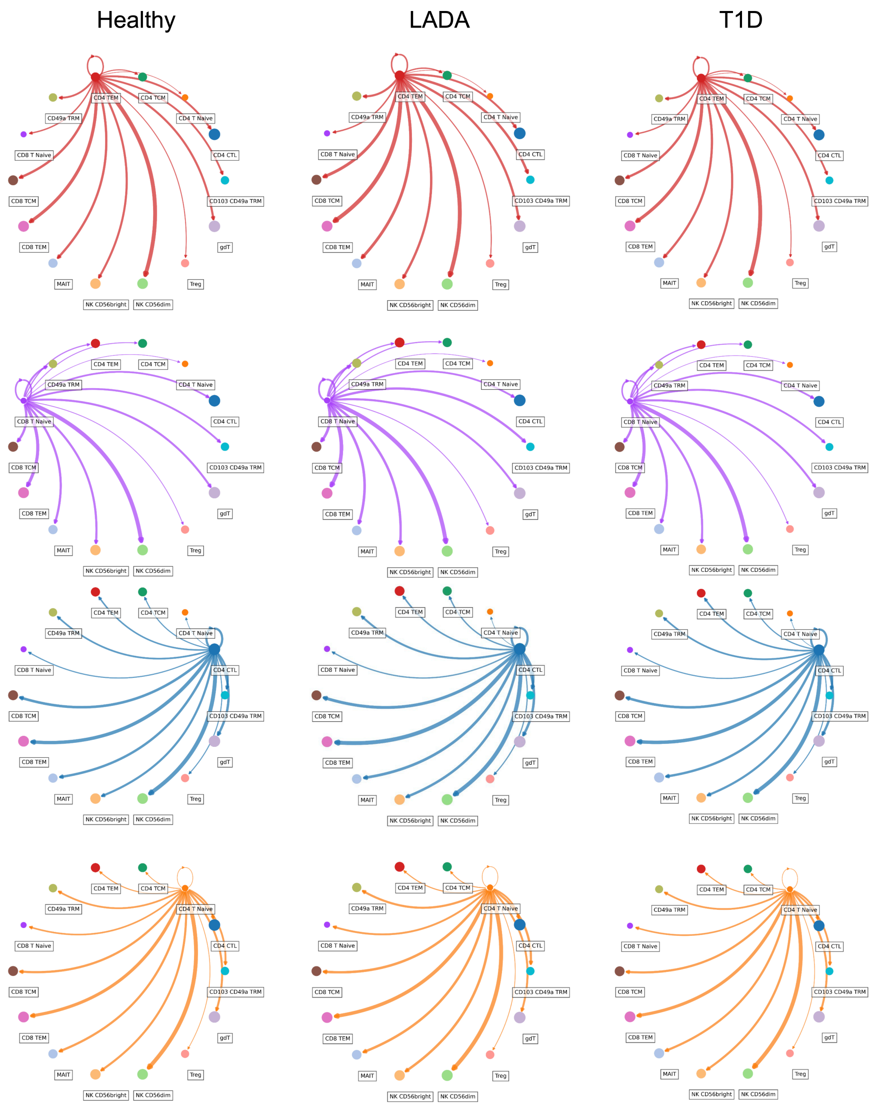

Supplementary Figure 5. Ligand–receptor interaction counts between T and NK cell subtypes. Each arrow represents the number of ligand–receptor interactions between cells. The origin of the arrow indicates the source of ligands, while the arrowhead points to the cell population expressing receptors for the secreted ligands. This figure compares number ligand-receptor interactions of CD4+ effector memory T cells (TEM), CD8+ naïve T cells, CD4+ cytotoxic T cells (CTL) and CD4+ naïve T cells from healthy donors (Healthy) and patients with latent autoimmune diabetes mellitus (LADA) and type 1 diabetes (T1D).

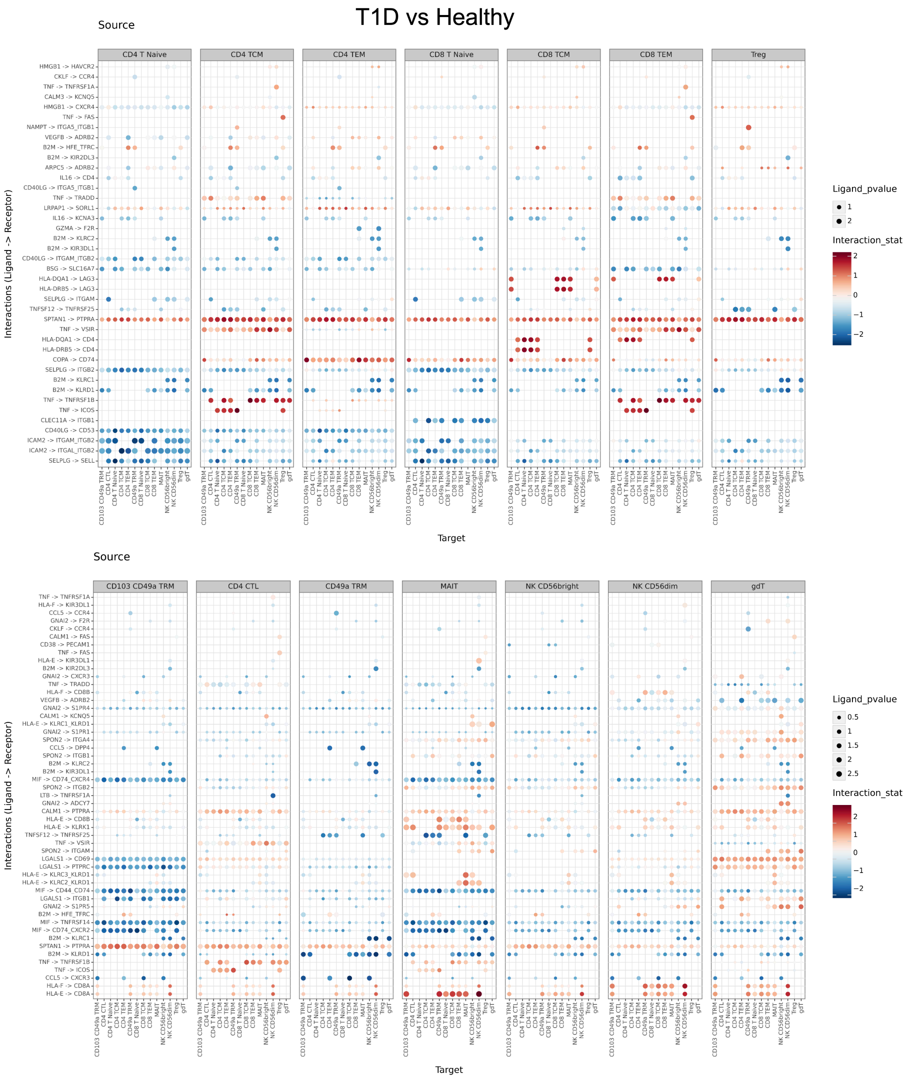

Supplementary Figure 6. Analysis of ligand–receptor interaction strength between T and NK cell subtypes in patients with type 1 diabetes (T1D) and healthy donors. Ligand–receptor pairs are shown on the left side of the figure. The cell types labeled at the top represent the sources of ligands, while the cell types labeled at the bottom express the receptor mRNA. Dot size indicates the p-value of the ligand–receptor interaction: the larger the dot, the smaller the p-value. All displayed interactions have a p-value < 0.05. The color of the dots represents the relative change in ligand–receptor interaction strength in cells from patients with type 1 diabetes (T1D) compared to cells from healthy donors. Dark red indicates an increase in interaction strength between cells in patients with type 1 diabetes (T1D) compared to healthy donors. Dark blue indicates a decrease in interaction strength between cells in patients with type 1 diabetes (T1D) compared to healthy donors.

LADA vs Healthy

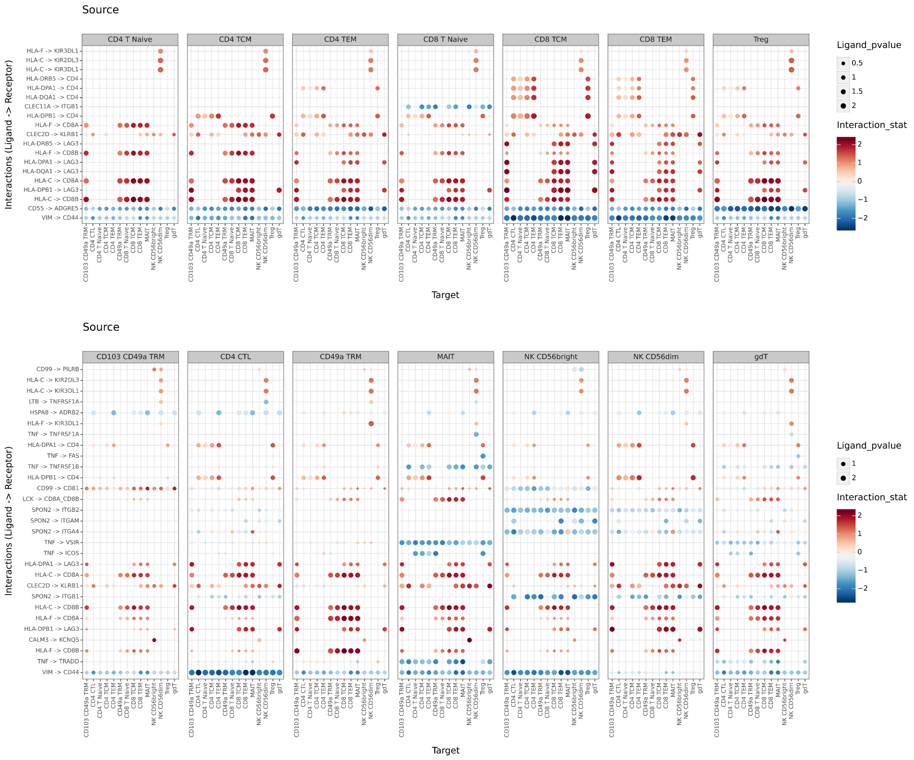

Supplementary Figure 7. Analysis of ligand–receptor interaction strength between T and NK cell subtypes in patients with latent autoimmune diabetes mellitus (LADA) and healthy donors. Ligand–receptor pairs are shown on the left side of the figure. The cell types labeled at the top represent the sources of ligands, while the cell types labeled at the bottom express the receptor mRNA. Dot size indicates the p-value of the ligand–receptor interaction: the larger the dot, the smaller the p-value. All displayed interactions have a p-value < 0.05. The color of the dots represents the relative change in ligand–receptor interaction strength in cells from patients with latent autoimmune diabetes mellitus (LADA) compared to cells from healthy donors. Dark red indicates an increase in interaction strength between cells in patients with latent autoimmune diabetes mellitus (LADA) compared to healthy donors. Dark blue indicates a decrease in interaction strength between cells in patients with latent autoimmune diabetes mellitus (LADA) compared to healthy donors.

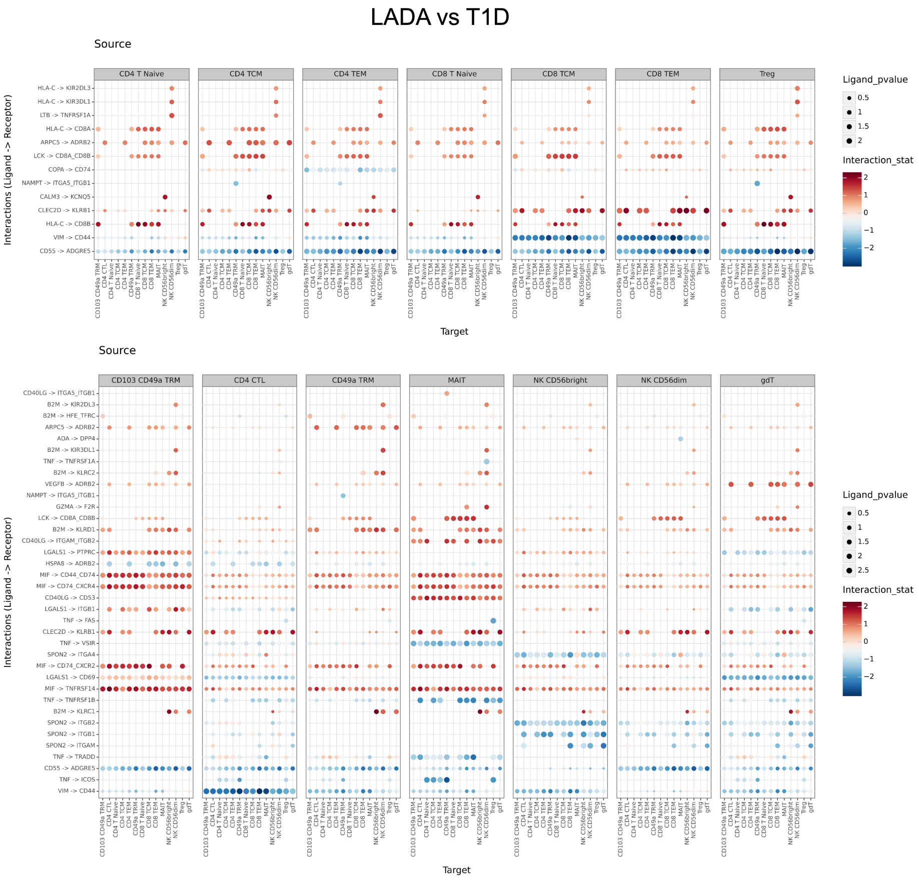

Supplementary Figure 8. Analysis of ligand–receptor interaction strength between T and NK cell subtypes in patients with latent autoimmune diabetes mellitus (LADA) and patients with type 1 diabetes (T1D). Ligand–receptor pairs are shown on the left side of the figure. The cell types labeled at the top represent the sources of ligands, while the cell types labeled at the bottom express the receptor mRNA. Dot size indicates the p-value of the ligand–receptor interaction: the larger the dot, the smaller the p-value. All displayed interactions have a p-value < 0.05. The color of the dots represents the relative change in ligand–receptor interaction strength in cells from patients with latent autoimmune diabetes mellitus (LADA) compared to cells from patients with type 1 diabetes (T1D). Dark red indicates an increase in interaction strength between cells in patients with latent autoimmune diabetes mellitus (LADA) compared to patients with type 1 diabetes (T1D). Dark blue indicates a decrease in interaction strength between cells in patients with latent autoimmune diabetes mellitus (LADA) compared to patients with type 1 diabetes (T1D).

Supplementary

Supplementary Table S1. Baseline clinical characteristics of study participants

**Parameter**	**T1D (*n* = 21)**	**LADA (*n* = 15)**	**Healthy Controls (*n* = 22)**	***P*-value**
Male sex, *n* (%)	10 (48%)	7 (47%)	11 (50%)	> 0.05
Age, years	26 [21; 31]	40 [34; 45]	33.5 [27; 42]	< 0.001
Disease duration, months	6 [2; 8]	38 [19; 53]	Not applicable	< 0.001
BMI, kg/m²	22.3 [20.1; 23.8]	23.0 [20.1; 23.9]	24.8 [20.6; 28.3]	0.214
HbA1c, %	7.8 [6.4; 10.4]	7.1 [6.6; 8.8]	5.1 [4.9; 5.4]	< 0.001

Legend: Statistical comparisons were performed using the chi-square test for categorical variables (male sex), Kruskal–Wallis test for continuous variables across all groups, and Mann–Whitney U test for pairwise comparison of disease duration between T1D and LADA.

Abbreviations: T1D, type 1 diabetes; LADA, latent autoimmune diabetes in adults; BMI, body mass index; HbA1c, glycated hemoglobin.
